# Distinction of *Plasmodium ovale wallikeri* and *Plasmodium ovale curtisi* using quantitative Polymerase Chain Reaction with High Resolution Melting revelation

**DOI:** 10.1038/s41598-017-18026-1

**Published:** 2018-01-10

**Authors:** V. Joste, C. Kamaliddin, E. Kendjo, V. Hubert, N. Argy, S. Houzé

**Affiliations:** 1 National French Malaria Reference Center, Bichat-Claude Bernard Hospital, 75018 Paris, France; 2 Parasitology and Mycology Laboratory, Bichat-Claude Bernard Hospital, APHP, 75018 Paris, France; 3 UMR216- MERIT, COMUE Sorbonne Paris Cité, Faculté de Pharmacie de Paris, Paris Descartes University, Paris, 75006 France; 4 National French Malaria Reference Center, Pitié Salpetrière hospital, 75013 Paris, France

## Abstract

*Plasmodium ovale curtisi* (*Poc*) and *Plasmodium ovale wallikeri* (*Pow*) have been described as two distinct species, only distinguishable by molecular methods such as PCR. Because of no well-defined endemic area and a variable clinical presentation as higher thrombocytopenia and nausea associated with *Pow* infection and asymptomatic forms of the pathology with *Poc* infection, rapid and specific identification of *Plasmodium ovale curtisi* and *Plasmodium ovale wallikeri* are needed. The aim of the study was to evaluate a new quantitative real-time PCR coupled with high resolution melting revelation (qPCR-HRM) for identification of both species. Results were compared with a nested-PCR, considered as a gold standard for *Pow* and *Poc* distinction. 356 samples including all human *Plasmodium* species at various parasitaemia were tested. The qPCR-HRM assay allowed *Poc* and *Pow* discrimination in 66 samples tested with a limit of detection evaluated at 1 parasite/µL. All these results were concordant with nested-PCR. Cross-reaction was absent with others blood parasites. The qPCR-HRM is a rapid and convenient technique to *Poc* and *Pow* distinction.

## Introduction

Malaria is triggered by an apicomplexan parasite, *Plasmodium* spp. 215 millions of cases and 429 000 deaths occurred in the world in 2015^1^. Six *Plasmodium* species infect humans: *Plasmodium falciparum* (*Pf*), *Plasmodium vivax* (*Pv*), *Plasmodium malariae* (*Pm*), *Plasmodium knowlesi* (*Pk*) and *Plasmodium ovale* spp (*P. ovale* spp) including *Plasmodium ovale curtisi* (*Poc*) and *Plasmodium ovale wallikeri* (*Pow*). Although malaria transmission was absent, France was the most exposed non endemic country for malaria^2^. Imported malaria cases diagnosed in France are reported to the National French Malaria Reference Center (FNMRC). In 2015, whereas *Pf* infection represented most of malaria cases (89%), *P. ovale* spp were involved in 6.8% of cases (4690 cases total)^3^, all contracted in Africa. Moreover, seven cases of *Plasmodium ovale* spp relapse (defined by malaria disease more than one year after the return from an endemic area) occurred in these patients (46% of the 15 observed relapses)^3^.

Malaria symptoms are nonspecific and the observation of thick and thin stained blood smears remained the reference method of diagnosis^4,5^. This technique is rapid and unexpensive, but required an experienced microscopist to ensure the quality of the result. However, species discrimination could be challenging, as *Poc* and *Pow* which cannot be reliably differentiated using this methodology.

Alternative ways to identify Plasmodium infection, such as Rapid Diagnosis Test (RDT) and PCR methods, have recently become available. Molecular biology diagnosis based methods are in constant expansion, in particular real-time methods. In the last decades, the malaria researcher community has developed several primers sets for species identification, to increase sensitivity in *Plasmodium* diagnosis.

First, nested-PCR methods with agarose gel revelation were developed. Probe based real-time PCR assays such as Taqman have been developed later on, and increased sensitivity and specificity. However, the sample processing and the technique itself are time consuming and cannot be applied in routine diagnosis procedures.

Up to 2010, scientists and biologist referred to *Poc* and *Pow* as *P. ovale* spp^6^. It is now admitted that *Poc* and *Pow* are two distinct species and not subspecies^7^. *Poc* and *Pow* are minor forms of malaria, however severe disease forms and deaths have been reported^8^. Differences in clinical implication, specific geographic repartition and response to antimalarial therapy might also exist between *Poc* and *Pow*, but the respective implication of each has not been completely studied yet. In addition, *Poc* and *Pow* physiopathology is unknown, especially mechanisms leading to the dormant form hypnozoite formation and relapses^9,10^. In fact, some differences in the latency period of the hypnozoites were highlighted^11^, higher thrombocytopenia and nausea with *Pow* was reported by another study^12^. Yet, microscopic examination of blood smear does not allow differentiation of the two species, even if a recent study described a lack of discernible Schüffner’s stippling in *Pow* samples^13^. Moreover, RDT efficiency was low and no differences in RDT positivity were found between *Poc* and *Pow* ^13^. Therefore, differentiation of *Poc* and *Pow* remains challenging, and the development of convenient tools to differentiate *Poc* and *Pow* is crucial to investigate the burden on malaria infections of each species, as well as their clinical implication.

*Poc* and *Pow* can be differentiated based on differences in their DNA sequences. Nested-PCR technique has been developed^14^ as well as qPCR protocols^15–18^. However, nested-PCR techniques are time-consuming requiring a two-step amplification followed by a PCR product migration on agarose gel. The entire process takes almost a day, and needs a dedicated environment for post-PCR products’ manipulation. Taqman probe based techniques are highly accurate and specific, but are expensive.

Here, we developed a new qPCR technique with High Resolution melting (HRM) (qPCR-HRM) revelation with the aim to reliably differentiate *Pow* and *Poc*, using samples from patients with imported malaria received at the FNMRC.

## Results

Of the 356 samples were included in the study period, 25 were negative for *Plasmodium* infection and 331 were positive according to qPCR-Taqman results (Supplementary Table S1): 219 were positive for *Pf*, 69 Po (subsequently identified as 33 *Pow*, and 36 *Poc* with nested PCR), 20 for *Pm*, 17 for *Pv*. 6 samples present mixed infection: 4*P. ovale* spp + *Pf* (subsequently identified for two *P. ovale* spp as *Poc*), 1*P. ovale* spp (subsequently identified as *Poc*) + *Pm* and *1P. ovale* spp + *Pm*. In microscopy, among 343 observed by microscopy, we detected 46 negative, 6 *Plasmodium* spp, 182 *Pf*, 20 *Pm*, 70 *P. ovale* spp, 17*Pv*, 1 *Pf* + *Pm*, 1 Pf + *P. ovale* spp.

For specificity analysis, we also included 6 other parasitological positive samples from blood parasites (*Toxoplasma gondii*, *Leishmania infantum*, *Loa loa*, *Onchocerca volvulus*, *Wuchereria bancrofti* and *Babesia divergens*).

### qPCR-HRM development

#### *In silico* prevision

*In silico* alignment was performed from predictive PCR product using the primers chosen for qPCR-HRM analysis. Plasmo-1F and Plasmo-2R primers amplify a 160-based pair (bp) and 157 bp sequence for *Poc* and *Pow* respectively. As a sequence variation in predictive PCR product for *Poc* and *Pow* was observed, HRM reaction could highlight a difference between both species. Using the specific uMELT online tool for PCR product melting temperature (Tm) analysis, predicting melting temperature from the HRM phase based on an algorithm analyzing DNA bounds strength, Tm could be predicted for the amplified sequences (77.4 °C for *Pow* and 76.8 °C for *Poc*). Predicted Tm as well as theorical derivated melting curves for other *Plasmodium* species are displayed in Supplementary Figure S2.

#### Distinction of P. ovale wallikeri and Plasmodium ovale curtisi

Among the 69*P. ovale* spp samples confirmed by qPCR-Taqman, Tm values for each species were reproducible (see Table 1 for average Tm and interquartile interval for *Poc* and *Pow*). 32 *Pow* and 34 *Poc* were identified by qPCR-HRM and these results were compared with nested-PCR followed by an electrophoresis in agarose gel and species were determined with 100% of concordance (Supplementary Table S1). However, by qPCR-HRM, 3 samples were misidentified as *Plasmodium malariae*: two were *Poc* and one was *Pow* by nested-PCR.

**Table 1:** Median melting temperature and interquartile range for each species.

	Pf	Pv	Pm	Pow	Poc	Pk
Median Tm1+/−interquartile range	72.892 +/− 0.100 (n = 208)	74.798 +/− 0.100 (n = 17)	73.926 +/− 0.179 (n = 18)	71.165 +/− 0.2285 (n = 31)	70.529 +/− 0.142 (n = 34)	75.289 (n = 2)
Median Tm2+/−interquartile range	75.396 +/− 0.112 (n = 210)			73.387 +/− 0.14725 (n = 33)	73.750 +/− 0.099 (n = 36)	

We considered qPCR-Taqman (for *Pf*, *Pv*, *Pm* and *P. ovale* spp) and nested-PCR (for *Poc* and *Pow*) as the reference for species determination.

**Figure 1:**
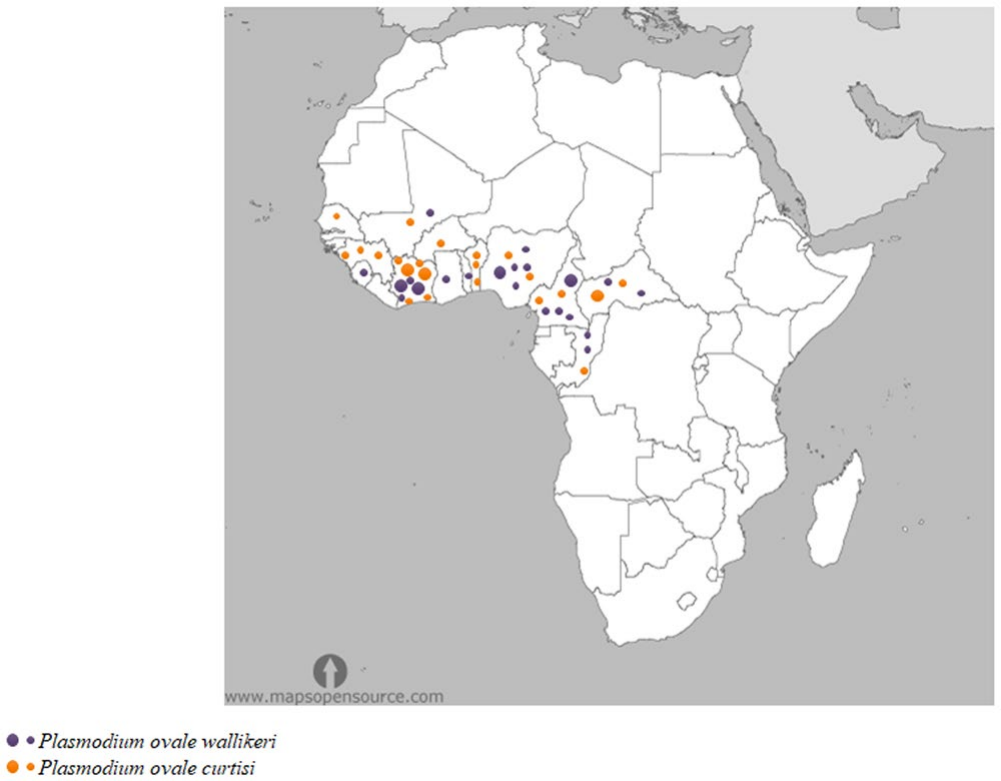
Map representing the origins of *P. ovale* spp samples included in the study. Orange represent *Poc* and purple *Pow*. Sign size is proportional to the number of samples from a given area. The smaller sign represents one sample, and the bigger one five samples. This map is modified from this URL: http://www.mapsopensource.com/africa-countries-outline-map-black-and-white.html whice provided open source map with no permission required (https://creativecommons.org/licenses/by/3.0/).

Specific geographic repartition of *Poc* and *Pow* in the included samples is presented in Fig. 1 and in Supplementary Table S1. *Pow* seems to be overrepresented in Cameroon whereas *Poc* seems to be overrepresented in Central African Republic. *Poc* and *Pow* are co-endemic in Ivory Coast, Cameroon, Central African Republic, Mali, Congo.

#### Mixed artificial samples with both *Poc* and *Pow*

Both *Poc* and *Pow* were distinguished in artificial samples, except when *Pow* was present in a strong majority (ratio 80% *Pow*/20% *Poc* and 90% *Pow*/10% *Poc*). Derivative melt curves of those samples are presented in Supplementary Figure S3. Same conclusions were made for *Pf*, *Pv*. *Pm*-*Po* mixed infections which could not be detected in mixed artificial samples.

### qPCR-HRM characteristics

#### Repeatability and reproducibility

Repeatability was evaluated for *Poc* and *Pow* using three different parasite’s densities. Repeatability was between 0.38 standard deviation (SD) and 0.49SD of Ct for *Poc* and, between 0.55 to 1.26 SD of Ct for *Pow*. Reproducibility was not evaluated as the assay was not quantitative. However, comparing the Tm for 10 samples on 10 distinct reactions, we observed a constant Tm in the different assay. Regarding *Poc*, coefficient variation was between 0.18 to 0.25% (70.90+/−0.132 to 70.90 +/− 0.162) for Tm1 and between 0.17 to 0.54% (73.98 +/− 0.13 °C to 74.31 +/− 0.405 °C) for Tm2. Regarding *Pow*, coefficient variation for Tm1 was between 0.20 to 0.47% (71.446 +/− 0.144 °C to 41.434 +/− 0.314 °C) and between 0.19 to 0.24% (73.708 +/− 0.144 °C to 73.834 +/− 0.174 °C) for Tm2.

#### Sensitivity and specificity

LOD was the lowest parasite concentration that had repeated positive signal detection with qPCR-HRM^19^ and was evaluated at 1 parasite/µL for both *Poc* and *Pow*.

No cross-reaction with *Toxoplasma gondii*, *Leishmania infantum*, *Loa loa*, *Onchocerca volvulus*, *Wuchereria bancrofti*, and *Babesia divergens* was detected with the qPCR-HRM technique. We observed significant amplification for *Toxoplasma gondii* and *Wuchereria bancrofti* but none of the Tm were specific to any *Plasmodium* (Supplementary Table S4).

**Figure 2:**
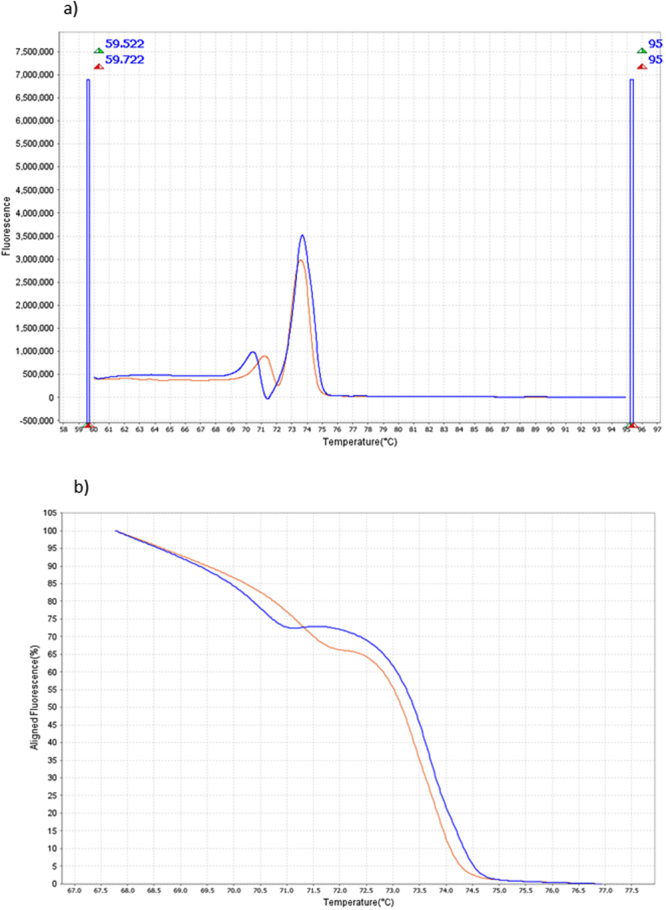
*Plasmodium ovale wallikeri* and *Plasmodium ovale curtisi* in derivative (**a**) and aligned melt curves (**b**) obtained after HRM phase in Viia7. X axis represents the temperature (°C). Y axis represent the fluorescence (derivative melt curve, **a**) or the aligned flurorescence (aligned melt curves, **b**). *Plasmodium ovale curtisi* profile is displayed in blue, and *Plasmodium ovale wallikeri* is displayed in orange.

As *in silico* prevision for the ability of the qPCR-HRM to distinguish *P. ovale* spp from other *Plasmodium* species, we applied the qPCR-HRM on 356 *Plasmodium* positive samples to confirm experimentally *in silico* data. Concordance between the qPCR-HRM and the qPCR-Taqman FTD Malaria Differentiation was observed for 336 samples (93.6%). Among the 23 (6.4%) discordant samples, there were with qPCR-HRM 11 false negative (sensitivity = 97%), 5 undetected species mixed (only one of both species detected), and 7 false positive results with crossing reactions between *Pf* and *Pv* (2 cases), *P. ovale* spp and *Pm* (3 cases), *Pm* and *Pv* (2 cases). No false positive were reported (specificity of 100%). We could distinguish *Plasmodium* species because *Pf* and *P. ovale* spp displayed a profile pattern with two specific Tm (Table 1) whereas melting curves obtained with samples positive for *Pm*, *Pv* and *Pk* displayed a profile with one specific Tm. *Poc* and *Pow* melting curves derivative plot showed two distinct profiles with Tms specific of each (70.529 °C and 73.750 °C for *Poc*, and 71.165 °C and 73.387 °C for *Pow*) (Fig. 2). In our batch of sample, we described three *P*. ovale spp with one remaining Tm (2 *Poc* and 1 *Pow*) with no relation with the Ct (mean Ct equal to 25.755 +/− 2.188).

All isolated species, except *Pm*, are in good concordance between qPCR-HRM and qPCR-Taqman (Table 2).

**Table 2:** Comparison between qPCR-Taqman, nested-PCR and qPCR-HRM results, by species, alone or in association.

qPCR-Taqman/Nested-PCR	qPCR-HRM								
	*Pf*	*Pv*	*Pm*	*Poc*	*Pow*	*Pf* + *Po*	*Pm* + *Po*	Negative	Total
*Pf*	**209**	1	0	0	0	0	0	9	219
*Pv*	1	**16**	0	0	0	0	0	0	17
*Pm*	0	2	**16**	0	0	0	0	2	20
*Poc*	0	0	2	**34**	0	0	0	0	36
*Pow*	0	0	1	**0**	32	0	0	0	33
*Pf* + *Po*	3	0	0	0	**0**	**1**	0	0	4
*Pm* + *Po*	0	0	1	1	0	0	**0**	0	2
Negative	0	0	0	0	0	0	0	**25**	25
Total	213	19	20	35	32	1	0	36	356

## Discussion

The existence of the two species *Poc* and *Pow* has been known since 2010, but has currently no impact on clinical care of the patient. It is not possible to differentiate them by optical microscopy. However, some clinical and biological differences have been noticed, such as higher proportion of thrombocytopenia and nausea with *Pow* ^12^. It also appears that *Poc* is more frequently asymptomatic^20^. For these reasons, a cost-effective qPCR-HRM method was developed for the differentiation of *Poc* and *Pow* species.

qPCR-HRM allowed us to discriminate *Poc* and *Pow* in confirmed *P. ovale* spp positive samples. To our knowledge, this is the first qPCR-HRM that allows *Poc* and *Pow* discrimination. LOD is similar to other qPCR-HRM already published^21^. Repeatability and reproducibility are really satisfying. From selected primer already published^22^, 18S RNA amplicons were aligned with the chosen primer to ensure the sequences divergence, and therefore the Tm variation, before setting the qPCR-HRM assay. The sequence presents noticeable variation between the *Pow* and *Poc* (98% consistency), and each parasites contains four to eight copies guaranteeing a good sensitivity to the PCR^23^ but this also complicates quantitative approaches. *In silico* prediction shows us different predictive Tm for *Poc* and *Pow* that convinced us to test Plasmo1-F and Plasmo2-R. In that *in silico* prediction, distinction between *P. ovale* spp and other *Plasmodium* species was also possible.

From 69 samples of *P. ovale* spp: 66 were identified by qPCR-HRM, and among those, concordance with nested PCR for species determination was perfect (100% concordance). This qPCR-HRM is fully efficient in *Poc* and *Pow* discrimination, after *P. ovale* spp confirmed identification: identification of other species than *Poc* or *Pow* don’t have to be considered because we demonstrated false identification. qPCR methods have already been published to distinguish species of *P. ovale* spp using specific probes in qPCR-Taqman^18^ but we present here an innovate way of PCR revelation. In our study we highlighted that *Poc* and *Pow* are co-endemic in some African countries such as Congo as previously described^15^. We demonstrated that *Poc* and *Pow* are also co-endemic in Ivory Coast, Cameroon, Central African Republic and Mali.

To assess the *in silico* data for the predicted *Poc* and *Pow* discrimination with other *Plasmodium* species infecting humans, qPCR-HRM was performed on other malaria positive samples. We couldn’t find the right specie in every sample. 18S RNA gene copies are located in 4 chromosomes among *Plasmodium* genome (chromosome 1, 5, 7 and 13). And for *Pf*, copies present 2 different sequences of 18S RNA gene. It explains the existence of two Tms as described by Kassaza *et al*.^24^ and shown in Supplementary Figure S2. We assume that the double Tm profile for *P. ovale* spp correspond also to genomic sequence variation (Fig. 2). *Pm* and *Pv* exhibit one specific Tm, with a difference in Tm of at least 0,5 °C between the two species. We tested two samples of *Pk* to assess the specificity of *Po* detection because *P. ovale* spp and *Pk* are co-endemic in Asia. Distinction of *Pk* and *P. ovale* spp was possible in all cases.

We reported 23 discordances in 356 samples (6.4%) in our data set between qPCR-HRM and qPCR-Taqman (FTD Malaria Diagnosis). There were eleven false negative results (nine for *Pf* and two for *Pm*) and discordances in species determination. We didn’t detect species combination in clinical samples whereas it was possible in artificial sample calibrated in laboratory, related to the difficulty to reveal the minor species. But, according to FNMRC data, *P. ovale* spp is rarely present in *Plasmodium* coinfection (3% in 2016)^3^ and co-infection malaria infections appear rare in import malaria (less than 1% of malaria infections in 2014 in United States^25^).

The rationale behind the development of our qPCR-HRM method was cost-efficiency for separating minor DNA sequence variation. It also provides an affordable molecular tool for laboratories, since only standard primers and protocols are required, and a same mix and protocol allows several analyses. qPCR-HRM allows examination of mutations in 400 bases pairs fragment whereas probe-based qPCR assays examine only twenty bases pairs fragment, are more expensive and present technical pitfalls which must be considered in large scale studies (e.g. probe conservation). Compared to nested-PCR, qPCR-HRM is faster, easier to interpret and limit experimental errors.

This qPCR-HRM allowed us to distinguish *Poc* and *Pow* in 100% cases. However, the technique does present some limits, especially for *Pm* with a risk of misdiagnosis. Here, some technical improvements could be evaluated, such as Mutant Allele Amplification Bias (MAAB)^26^ or cold-PCR^27^ to discriminate the minor species in mixed infection, using the qPCR-HRM method we have described here. Those technical innovations should be conducted in the context of *Plasmodium* genomes and may be used to detect minor species in a mixed sample. Additional solutions may be considered, such as an increase DNA concentration to lower sensitivity threshold, or increase time of PCR (with a higher risk of false positive).

To conclude, we developed a qPCR-HRM for *Poc* and *Pow* discrimination, that can be used in epidemiological studies on the geographical distribution of each species or in studies aimed at detecting differences in clinical, prognostic and sensitivity to anti-malaria drugs. In general, this will facilitate a better understanding of the biological differences between *Poc* and *Pow* and their incidence in pathology.

## Materials and Methods

### Sample collection

Between January 2015 and August 2015, fresh blood samples collected on EDTA received in the FNMRC for expertise from patients were included. We also tested 2 *Pk* positive samples.

No specific consent was required because of, in coordination with Sante Publique France organization for the care and surveillance of malaria, the parasitological data were collected in the FNMRC database and analyzed in accordance with the common public health mission of all French National Reference Centers. The study of the biological samples obtained in the medical care context was considered as a non-interventional research (article L1221-1.1 of the French public health code) only requiring the non-opposition of the patient during sampling (article L1211-2 of the French public health code). All data collected were anonymized before analysis.

### DNA extraction

DNA was extracted from a sample of 200 µL of whole blood sample using Magnapure^®^ (Roche diagnosis, Bale, Switzerland) and was eluted in 100 µL of buffer following manufacturer’s instructions. DNA was stored at −20 °C until species analyses.

### Species identification

On each included sample, microscopy diagnosis and qPCR-Taqman for *Pf*, *Pv*, *Pm*, *P. ovale* spp determination. On positive samples for *P. ovale* spp with qPCR-Taqman, *Poc* and *Pow* distinction was determined by nested-PCR. We then performed qPCR-HRM on each sample.

### Microscopic investigation

Thick blood smears were considered positive if one or more malaria parasites were visualized and negative if no parasites were detected after examining 1000 white blood cells. Thin blood smears were read by two distinct operators. Parasite species was determined using thin blood smears. Parasitaemia was expressed in parasite density (parasites/µL) following this formula:

1 $${\rm{parasite}}\,{\rm{density}}={\rm{45000}}\ast {\rm{parasitaemia}}\,(\mathrm{parasites}/\mu {\rm{L}}).$$

### qPCR-Taqman method

Species identification was confirmed by qPCR-Taqman (FTD Malaria, Fast-track Diagnostics, Launch diagnostics®) following manufacturer’s instruction. All samples were run in qPCR-Taqman.

### Nested-PCR for *P. knowlesi* determination

*Pk* infection was confirmed by PCR as described by Singh *et al*.^28^.

### Nested-PCR for determination of *Poc* and *Pow*

All *P. ovale* spp positive samples in qPCR-Taqman were run in nested-PCR. We used nested-PCR previously described for *Poc* and *Pow* determination^29^. The first PCR employed primer pair rPLU1 (5’-TCA-AAG-ATT-AAG-CCA-TGC-AAG-TGA-3′) and rPLU5 (5′-CCT-GTT-GTT-GCC-TTA-AAC-TTC-3′) primers for a *Plasmodium* genus reaction^30^. The second PCR reaction was performed with primer pair rOVA1 (5′-ATC-TCT-TTT-GCT-ATT-TTT-TAG-TAT-TGG-AGA-3′)/rOVA2 (5′-GGA-AAA-GGA-CAC-ATT-AAT-TGT-ATC-CTA-GTG-3′) for *Poc* identification^31^ and primer pair rOVA1v (5′-ATC-TCC-TTT-ACT-TTT-TGT-ACT-GGA-GA-3′)/rOVA2v (5′-GGA-AAA-GGA-CAC-TAT-AAT-GTA-TCC-TAA-TA-3′) for *Pow* identification^14,29^. PCR products were visualized on 2% agarose gels stained with Gelred^®^ (10000X, Biotium, inc).

### qPCR-HRM development

**Figure 3:**
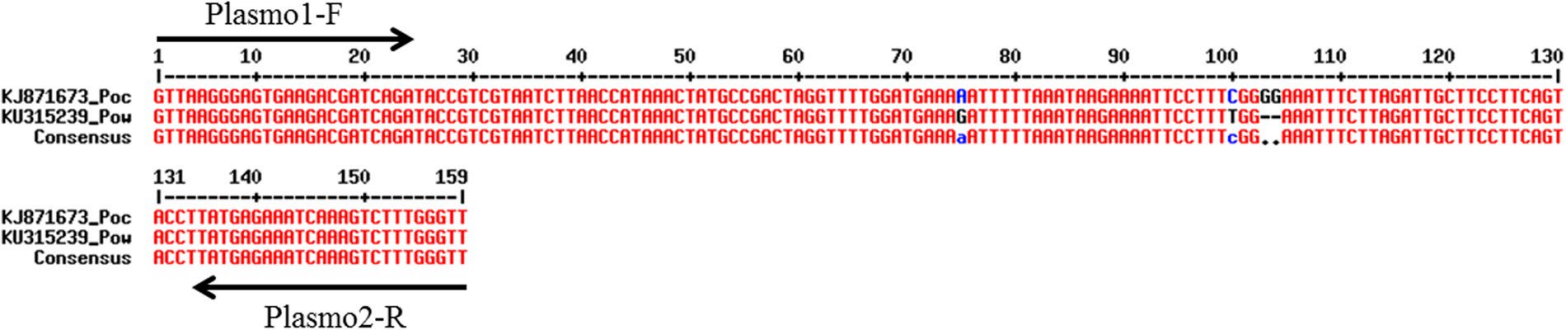
Alignement of 18S RNA amplified sequence from *Plasmodium ovale curtisi* and *Plasmodium ovale wallikeri* using MultiAlin. Primer position are displayed on 18S RNA gene sequence (Plasmo1-F and Plasmo2-R). The insertion of two Guanine in position 103 and 104 allows species differentiation using HRM detection.

Primers used in the qPCR-HRM were Plasmo1-F (5′-GTT-AAG-GGA-GTG-AAG-ACG-ATC-AGA-3′) and Plasmo2-R (5′-AAC-CCA-AAG-ACT-TTG-ATT-TCT-CAT-AA-3′), targeting the 18S RNA gene which contains both highly conserved and variable regions in all *Plasmodium* species infecting humans, previously described by Rougemont *et al*.^22,32^ (Fig. 3).

To set up a one-step PCR method for *P. ovale* spp species determination, differences in the PCR products for a given set of PCR primers were investigated to know if they could present enough differences to be noticeable with a HRM based PCR product detection. To this end, we first aligned the PCR products from the *in silico* PCR, using the multialin online tool (http://multalin.toulouse.inra.fr/multalin/)^33^. Then, we performed a melting temperature simulation, using uMELT online tool (available at https://www.dna.utah.edu/umelt/umb.php), to assess if the differences observed in the PCR products would be sufficient or not to distinguish *Poc* and *Pow* using HRM detection^34^ and to distinguish *P. ovale* spp from other *Plasmodium* species.

### PCR reaction

The PCR mixture included 0.3 µM of each primer, 12.5 µL of MeltDoctor® HRM master mix (Life Technologies, Carlsbad, USA), 5 µL of DNA extract and water for a final volume of 20 µL. The thermal profile used for qPCR was as follows: 10 min initial denaturation at 95 °C; 40 cycles of 10 s at 95 °C denaturation and 1 min at 60 °C; 10 s at 95 °C followed by 1 min at 60 °C before HRM phase. HRM phase consisted on a 0.025 °C/s temperature increase. Melt curve plot was generated and analysed with Viia 7 software (Applied Biosystems) to determine Tm.

### Mixed artificial samplesh

Artificial samples were performed with both *Poc* and *Pow* to determine the ability of qPCR-HRM to differentiate *Poc* and *Pow* in the same isolate. We prepared 9 artificial samples with various ratio of *Poc* and *Pow* (9/1; 8/2; 7/3; 6/4; 5/5; for both). Initial parasite density of *Poc* and *Pow* was 3000 parasites/µL and 6000 parasites/µl. Similar artificial samples (*Poc* and *Pow* with other *Plasmodium* species in the same isolate) were prepared for *Pf*, *Pv* and *Pm*.

### qPCR-HRM performance

#### Repeatability and reproducibility

Repeatability was determined by using a unique sample thirty times in the same run for three different parasite density for both *Poc* and *Pow* at 3200 parasites/µL, 640 parasites/µL and 64 parasites/µL. We calculated standard deviation of Ct for repeatability as recommended in the MIQE guidelines^19^. We evaluated reproducibility as concordance of Tm between different run for the same isolate at three different parasite density for *Poc* and *Pow* (6400 parasites/µL, 640 parasites/µL and 64 parasites/µL).

#### Sensitivity and specificity

A serial sample dilution was performed to obtain eleven points ranging from 4000 to 1 parasites/µL from one *Poc* and one *Pow* positive sample, to determine the limit of detection (LOD). We calculated sensitivity of the method following this formula:

2 $${\rm{Sensitivity}}=(1-\frac{False\,Negative}{Total\,samples})\ast 100.$$

Ct amplification and melting curve were used to define LOD and samples were considered as negative if no specific Tm was observed.

Specificity was evaluated in two different way. First, we tested *Toxoplasma gondii*, *Leishmania infantum*, *Loa loa*, *Onchocerca volvulus*, *Wuchereria bancrofti*, and *Babesia divergens* patient positive blood samples from Bichat’s hospital to prove the existence of *Poc* and *Pow* specific Tm. Second, we evaluated the ability of the qPCR-HRM of distinguish *P. ovale* spp from other *Plasmodium* species in clinical samples.

### Ethic approval

Participants’ consent was not required since samples were already collected for other medical purposes, and according to French legislations on non-interventional research.

## Electronic supplementary material

Supplementary Dataset 1
