# Supplementary material for: Distinction of Plasmodium ovale wallikeri and Plasmodium ovale curtisi using quantitative Polymerase Chain Reaction with High Resolution Melting revelation
Source: Sci Rep. 2018 Jan 10;8:300. doi: 10.1038/s41598-017-18026-1 (PMC5762660; doi:10.1038/s41598-017-18026-1)
Supplement: Supplementary file 1 — Supplementary Dataset 1 [file 41598_2017_18026_MOESM1_ESM.doc]

Distinction of *Plasmodium ovale wallikeri* and *Plasmodium ovale curtisi* using quantitative Polymerase Chain Reaction with High Resolution Melting revelation

Authors: Joste V^1,2^, Kamaliddin C^1,3^, Kendjo E^1,4^, Hubert V^1,3^, Argy N^1,2,3^, Houzé S^1,2,3^

Authors’ affiliation :

1. National French Malaria Reference Center, Bichat-Claude Bernard Hospital, 75018 Paris, France
2. Parasitology and Mycology Laboratory, Bichat-Claude Bernard Hospital, APHP, 75018, Paris, France
3. UMR216- MERIT, COMUE Sorbonne Paris Cité, Faculté de Pharmacie de Paris, Paris Descartes University, Paris 75006, France
4. National French Malaria Reference Center, Pitié Salpetrière hospital, 75013 Paris, France

Corresponding author: Valentin Joste, valentin.joste@aphp.fr

| **­­Sample** | **Ct in qPCR-HRM** | **Tm1** | **Tm2** | **Tm3** | **qPCR-HRM determination** | **qPCR-Taqman determination** | **Nested determination** | **Microscopic determination** | **Country of contamination** |
| --- | --- | --- | --- | --- | --- | --- | --- | --- | --- |
| 1 | Undetermined |  |  |  | Negative | Negative |  | Negative |  |
| 2 | Undetermined |  |  |  | Negative | Negative |  | Positive |  |
| 3 | Undetermined |  |  |  | Negative | Negative |  | Negative |  |
| 4 | Undetermined |  |  |  | Negative | Negative |  | Negative |  |
| 5 | Undetermined |  |  |  | Negative | Negative |  | Negative |  |
| 6 | Undetermined |  |  |  | Negative | Negative |  | Negative |  |
| 7 | Undetermined |  |  |  | Negative | Negative |  | Negative |  |
| 8 | Undetermined |  |  |  | Negative | Negative |  | Negative |  |
| 9 | Undetermined |  |  |  | Negative | Negative |  | Negative |  |
| 10 | Undetermined |  |  |  | Negative | Negative |  | *Pf* |  |
| 11 | Undetermined |  |  |  | Negative | Negative |  | Negative |  |
| 12 | Undetermined |  |  |  | Negative | Negative |  | *Pf* |  |
| 13 | Undetermined |  |  |  | Negative | Negative |  | Negative |  |
| 14 | Undetermined |  |  |  | Negative | Negative |  | Negative |  |
| 15 | Undetermined |  |  |  | Negative | Negative |  | Negative |  |
| 16 | Undetermined |  |  |  | Negative | Negative |  | Negative |  |
| 17 | Undetermined |  |  |  | Negative | Negative |  | Negative |  |
| 18 | 39.923 | 79.660 |  |  | Negative | Negative |  | Negative |  |
| 19 | 39.066 | 79.801 |  |  | Negative | Negative |  | Negative |  |
| 20 | Undetermined |  |  |  | Negative | Negative |  | Negative |  |
| 21 | Undetermined |  |  |  | Negative | Negative |  | Negative |  |
| 22 | 29.713 | 78.021 |  |  | Negative | Negative |  | Negative |  |
| 23 | Undetermined |  |  |  | Negative | Negative |  | Negative |  |
| 24 | Undetermined |  |  |  | Negative | Negative |  | Negative |  |
| 25 | Undetermined |  |  |  | Negative | Negative |  | Negative |  |
| 26 | Undetermined |  |  |  | Negative | *Pf* |  | Negative |  |
| 27 | Undetermined |  |  |  | Negative | *Pf* |  | Negative |  |
| 28 | Undetermined |  |  |  | Negative | *Pf* |  | *Pf* |  |
| 29 | Undetermined |  |  |  | Negative | *Pf* |  | Negative |  |
| 30 | Undetermined |  |  |  | Negative | *Pf* |  | *Pf* |  |
| 31 | Undetermined |  |  |  | Negative | *Pf* |  | *Pf* |  |
| 32 | Undetermined |  |  |  | Negative | *Pf* |  | *Pf* |  |
| 33 | Undetermined |  |  |  | Negative | *Pf* |  | Negative |  |
| 34 | 30.684 | 75.200 | 72.750 | 71.175 | *Pf* | *Pf* |  | *Pf* |  |
| 35 | 38.301 | 75.311 |  |  | *Pf* | *Pf* |  | *Pf* |  |
| 36 | 39.487 | 75.467 |  |  | *Pf* | *Pf* |  | Negative |  |
| 37 | 38.103 | 72.948 | 80.450 |  | *Pf* | *Pf* |  | Negative |  |
| 38 | 28.437 | 73.052 | 75.568 |  | *Pf* | *Pf* |  | *Pf* |  |
| 39 | 31.550 | 73.027 | 75.527 |  | *Pf* | *Pf* |  | *Pf* |  |
| 40 | 29.380 | 73.007 | 75.507 |  | *Pf* | *Pf* |  | *Pf* |  |
| 41 | 34.991 | 72.981 | 75.504 |  | *Pf* | *Pf* |  | *Pf* |  |
| 42 | 34.895 | 72.930 | 75.480 |  | *Pf* | *Pf* |  | Negative |  |
| 43 | 36.066 | 72.962 | 75.474 |  | *Pf* | *Pf* |  | *Pf* |  |
| 44 | 28.625 | 72.941 | 75.464 |  | *Pf* | *Pf* |  | *Pf* |  |
| 45 | 32.849 | 72.938 | 75.461 |  | *Pf* | *Pf* |  | *Pf* |  |
| 46 | 25.604 | 72.938 | 75.461 |  | *Pf* | *Pf* |  | *Pf* |  |
| 47 | 28.520 | 72.937 | 75.460 |  | *Pf* | *Pf* |  | *Pf* |  |
| 48 | 30.692 | 73.014 | 75.450 |  | *Pf* | *Pf* |  | *Pf* |  |
| 49 | 26.975 | 72.918 | 75.441 |  | *Pf* | *Pf* |  | *Pf* |  |
| 50 | 28.127 | 72.939 | 75.437 |  | *Pf* | *Pf* |  | *Pf* |  |
| 51 | 29.412 | 72.938 | 75.436 |  | *Pf* | *Pf* |  | *Pf* |  |
| 52 | 27.779 | 72.927 | 75.428 |  | *Pf* | *Pf* |  | *Pf* |  |
| 53 | 27.171 | 72.962 | 75.423 | 70.501 | *Pf* | *Pf* |  | *Pf* |  |
| 54 | 33.676 | 72.922 | 75.423 |  | *Pf* | *Pf* |  | *Pf* |  |
| 55 | 34.086 | 72.938 | 75.411 | 79.733 | *Pf* | *Pf* |  | Negative |  |
| 56 | 29.424 | 72.875 | 75.400 |  | *Pf* | *Pf* |  | *Pf* |  |
| 57 | 32.500 | 72.868 | 75.391 |  | *Pf* | *Pf* |  | *Pf* |  |
| 58 | 26.308 | 72.890 | 75.388 |  | *Pf* | *Pf* |  | *Pf* |  |
| 59 | 26.129 | 72.839 | 75.387 |  | *Pf* | *Pf* |  | *Pf* |  |
| 60 | 23.130 | 72.861 | 75.373 |  | *Pf* | *Pf* |  | Negative |  |
| 61 | 30.189 | 72.892 | 75.365 |  | *Pf* | *Pf* |  | *Pf* |  |
| 62 | 31.074 | 72.853 | 75.353 |  | *Pf* | *Pf* |  | *Pf* |  |
| 63 | 29.947 | 72.954 | 75.279 | 70.578 | *Pf* | *Pf* |  | *Pf* |  |
| 64 | 24.396 | 72.821 | 75.247 |  | *Pf* | *Pf* |  | *Pf* |  |
| 65 | 31.284 | 72.765 | 75.238 |  | *Pf* | *Pf* |  | *Pf* |  |
| 66 | 22.376 | 72.826 | 75.226 |  | *Pf* | *Pf* |  | No result |  |
| 67 | 29.617 | 72.901 | 75.226 |  | *Pf* | *Pf* |  | *Pf* |  |
| 68 | 25.290 | 72.846 | 75.147 |  | *Pf* | *Pf* |  | *Pf* |  |
| 69 | 21.358 | 72.875 | 74.794 |  | *Pf* | *Pf* |  | No result |  |
| 70 | 26.438 | 75.836 | 73.363 |  | *Pf* | *Pf* |  | No result |  |
| 71 | 30.000 | 75.713 | 73.215 | 71.516 | *Pf* | *Pf* |  | *Pf* |  |
| 72 | 27.984 | 75.601 | 73.114 |  | *Pf* | *Pf* |  | *Pf* |  |
| 73 | 28.433 | 75.559 | 73.059 |  | *Pf* | *Pf* |  | *Pf* |  |
| 74 | 23.847 | 75.556 | 73.058 |  | *Pf* | *Pf* |  | *Pf* |  |
| 75 | 27.171 | 75.500 | 73.013 |  | *Pf* | *Pf* |  | *Pf* |  |
| 76 | 35.943 | 75.536 | 73.013 |  | *Pf* | *Pf* |  | Negative |  |
| 77 | 22.225 | 75.532 | 73.009 |  | *Pf* | *Pf* |  | *Pf* |  |
| 78 | 28.512 | 75.532 | 73.009 |  | *Pf* | *Pf* |  | *Pf* |  |
| 79 | 24.100 | 75.529 | 73.004 |  | *Pf* | *Pf* |  | *Pf* |  |
| 80 | 32.542 | 75.526 | 73.001 |  | *Pf* | *Pf* |  | Negative |  |
| 81 | 27.709 | 75.525 | 73.000 |  | *Pf* | *Pf* |  | *Pf* |  |
| 82 | 23.815 | 75.476 | 72.989 | 71.365 | *Pf* | *Pf* |  | *Pf* |  |
| 83 | 34.339 | 75.462 | 72.989 |  | *Pf* | *Pf* |  | Negative |  |
| 84 | 21.744 | 75.500 | 72.988 |  | *Pf* | *Pf* |  | *Pf* |  |
| 85 | 23.093 | 75.533 | 72.985 |  | *Pf* | *Pf* |  | *Pf* |  |
| 86 | 30.248 | 75.507 | 72.982 |  | *Pf* | *Pf* |  | *Pf* |  |
| 87 | 24.744 | 75.479 | 72.981 |  | *Pf* | *Pf* |  | *Pf* |  |
| 88 | 25.995 | 75.480 | 72.980 |  | *Pf* | *Pf* |  | *Pf* |  |
| 89 | 26.797 | 75.502 | 72.979 |  | *Pf* | *Pf* |  | *Pf* |  |
| 90 | 29.590 | 75.475 | 72.974 |  | *Pf* | *Pf* |  | *Pf* |  |
| 91 | 29.017 | 75.494 | 72.971 |  | *Pf* | *Pf* |  | *Pf* |  |
| 92 | 24.003 | 75.489 | 72.966 |  | *Pf* | *Pf* |  | *Pf* |  |
| 93 | 30.190 | 75.423 | 72.962 |  | *Pf* | *Pf* |  | *Pf* |  |
| 94 | 28.297 | 75.474 | 72.962 |  | *Pf* | *Pf* |  | *Pf* |  |
| 95 | 34.578 | 75.456 | 72.958 |  | *Pf* | *Pf* |  | Negative |  |
| 96 | 22.908 | 75.444 | 72.952 |  | *Pf* | *Pf* |  | No result |  |
| 97 | 31.958 | 75.477 | 72.952 |  | *Pf* | *Pf* |  | *Pf* |  |
| 98 | 28.203 | 75.416 | 72.943 |  | *Pf* | *Pf* |  | *Pf* |  |
| 99 | 31.733 | 75.403 | 72.942 |  | *Pf* | *Pf* |  | *Pf* |  |
| 100 | 31.963 | 75.414 | 72.941 |  | *Pf* | *Pf* |  | *Pf* |  |
| 101 | 30.992 | 75.452 | 72.940 |  | *Pf* | *Pf* |  | *Pf* |  |
| 102 | 23.459 | 75.549 | 73.049 |  | *Pf* | *Pf* |  | *Pf* |  |
| 103 | 31.022 | 75.411 | 72.938 |  | *Pf* | *Pf* |  | *Pf* |  |
| 104 | 30.284 | 75.411 | 72.938 |  | *Pf* | *pf* |  | *Pf* |  |
| 105 | 31.868 | 75.424 | 72.937 | 80.296 | *Pf* | *Pf* |  | *P spp* |  |
| 106 | 21.458 | 75.460 | 72.937 |  | *Pf* | *Pf* |  | *Pf Pm* |  |
| 107 | 32.956 | 75.372 | 72.936 |  | *Pf* | *Pf* |  | *Pf* |  |
| 108 | 32.160 | 75.447 | 72.935 |  | *Pf* | *Pf* |  | *Pf* |  |
| 109 | 32.128 | 75.472 | 72.935 |  | *Pf* | *Pf* |  | *Pf* |  |
| 110 | 32.201 | 75.458 | 72.935 |  | *Pf* | *Pf* |  | *Pf* |  |
| 111 | 33.311 | 75.456 | 72.931 | 79.732 | *Pf* | *Pf* |  | *Pf* |  |
| 112 | 28.169 | 75.430 | 72.930 |  | *Pf* | *Pf* |  | *Pf* |  |
| 113 | 28.530 | 75.444 | 72.927 |  | *Pf* | *Pf* |  | *Pf* |  |
| 114 | 34.615 | 75.452 | 72.927 |  | *Pf* | *Pf* |  | Negative |  |
| 115 | 30.623 | 75.418 | 72.926 |  | *Pf* | *Pf* |  | Negative |  |
| 116 | 22.000 | 75.418 | 72.926 |  | *Pf* | *Pf* |  | *Pf* |  |
| 117 | 28.877 | 75.426 | 72.926 |  | *Pf* | *Pf* |  | *Pf* |  |
| 118 | 28.922 | 75.424 | 72.924 |  | *Pf* | *Pf* |  | *Pf* |  |
| 119 | 34.396 | 75.391 | 72.924 | 79.203 | *Pf* | *Pf* |  | Negative |  |
| 120 | 30.247 | 75.399 | 72.923 |  | *Pf* | *Pf* |  | *Pf* |  |
| 121 | 32.655 | 75.422 | 72.921 |  | *Pf* | *Pf* |  | *Pf* |  |
| 122 | 28.107 | 75.376 | 72.915 | 71.215 | *Pf* | *Pf* |  | *Pf* |  |
| 123 | 28.748 | 75.426 | 72.914 |  | *Pf* | *Pf* |  | *Pf* |  |
| 124 | 29.503 | 75.424 | 72.912 |  | *Pf* | *Pf* |  | *Pf* |  |
| 125 | 29.543 | 75.449 | 72.912 |  | *Pf* | *Pf* |  | *Pf* |  |
| 126 | 29.871 | 75.435 | 72.912 |  | *Pf* | *Pf* |  | *Pf* |  |
| 127 | 30.199 | 75.373 | 72.911 |  | *Pf* | *Pf* |  | *Pf* |  |
| 128 | 31.759 | 75.347 | 72.911 |  | *Pf* | *Pf* |  | *Pf* |  |
| 129 | 15.842 | 75.420 | 72.903 |  | *Pf* | *Pf* |  | *Pf* |  |
| 130 | 24.103 | 75.369 | 72.902 |  | *Pf* | *Pf* |  | *Pf* |  |
| 131 | 26.766 | 75.394 | 72.902 |  | *Pf* | *Pf* |  | *Pf* |  |
| 132 | 23.186 | 75.394 | 72.902 |  | *Pf* | *Pf* |  | *Pf* |  |
| 133 | 25.383 | 75.418 | 72.901 |  | *Pf* | *Pf* |  | *Pf* |  |
| 134 | 26.850 | 75.399 | 72.899 |  | *Pf* | *Pf* |  | *Pf* |  |
| 135 | 29.977 | 75.424 | 72.899 |  | *Pf* | *Pf* |  | *Pf* |  |
| 136 | 27.309 | 75.399 | 72.898 |  | *Pf* | *Pf* |  | *Pf* |  |
| 137 | 23.511 | 75.373 | 72.897 |  | *Pf* | *Pf* |  | *Pf* |  |
| 138 | 24.000 | 75.388 | 72.897 |  | *Pf* | *Pf* |  | *Pf* |  |
| 139 | 27.769 | 75.393 | 72.895 |  | *Pf* | *Pf* |  | *Pf* |  |
| 140 | 27.780 | 75.390 | 72.892 |  | *Pf* | *Pf* |  | *Pf* |  |
| 141 | 30.858 | 75.362 | 72.889 |  | *Pf* | *Pf* |  | *Pf* |  |
| 142 | 38.484 | 75.437 | 72.889 | 79.135 | *Pf* | *Pf* |  | Negative |  |
| 143 | 29.168 | 75.349 | 72.888 |  | *Pf* | *Pf* |  | *Pf* |  |
| 144 | 32.991 | 75.374 | 72.888 |  | *Pf* | *Pf* |  | *Pf* |  |
| 145 | 31.002 | 75.425 | 72.888 |  | *Pf* | *Pf* |  | *Pf* |  |
| 146 | 30.250 | 75.386 | 72.888 |  | *Pf* | *Pf* |  | *Pf* |  |
| 147 | 31.735 | 75.399 | 72.887 |  | *Pf* | *Pf* |  | *Pf* |  |
| 148 | 29.721 | 75.425 | 72.887 |  | *Pf* | *Pf* |  | *Pf* |  |
| 149 | 33.856 | 75.410 | 72.887 |  | *Pf* | *Pf* |  | *Pf* |  |
| 150 | 29.787 | 75.424 | 72.887 |  | *Pf* | *Pf* |  | *Pf* |  |
| 151 | 25.632 | 75.398 | 72.886 |  | *Pf* | *Pf* |  | *Pf* |  |
| 152 | 22.097 | 75.397 | 72.885 |  | *Pf* | *Pf* |  | *Pf* |  |
| 153 | 26.053 | 75.397 | 72.885 |  | *Pf* | *Pf* |  | *Pf* |  |
| 154 | 26.649 | 75.422 | 72.885 |  | *Pf* | *Pf* |  | *Pf* |  |
| 155 | 20.363 | 75.375 | 72.875 |  | *Pf* | *Pf* |  | No result |  |
| 156 | 16.500 | 75.415 | 72.874 |  | *Pf* | *Pf* |  | *Pf* |  |
| 157 | 26.579 | 75.374 | 72.873 | 80.300 | *Pf* | *Pf* |  | *Pf* |  |
| 158 | 24.780 | 75.374 | 72.873 |  | *Pf* | *Pf* |  | *Pf* |  |
| 159 | 25.462 | 75.348 | 72.872 |  | *Pf* | *Pf* |  | *Pf* |  |
| 160 | 25.000 | 75.362 | 72.864 |  | *Pf* | *Pf* |  | *Pf* |  |
| 161 | 29.563 | 75.349 | 72.862 |  | *Pf* | *Pf* |  | *Pf* |  |
| 162 | 32.650 | 75.399 | 72.861 |  | *Pf* | *Pf* |  | Negative |  |
| 163 | 26.331 | 75.398 | 72.861 |  | *Pf* | *Pf* |  | *Pf* |  |
| 164 | 38.548 | 75.423 | 72.860 |  | *Pf* | *Pf* |  | Negative |  |
| 165 | 24.238 | 75.371 | 72.859 |  | *Pf* | *Pf* |  | *Pf* |  |
| 166 | 28.864 | 75.369 | 72.852 |  | *Pf* | *Pf* |  | *Pf* |  |
| 167 | 26.200 | 75.369 | 72.852 |  | *Pf* | *Pf* |  | *Pf* |  |
| 168 | 25.127 | 75.352 | 72.852 |  | *Pf* | *Pf* |  | *Pf* |  |
| 169 | 22.000 | 75.368 | 72.851 |  | *Pf* | *Pf* |  | *Pf* |  |
| 170 | 27.400 | 75.324 | 72.849 |  | *Pf* | *Pf* |  | *Pf* |  |
| 171 | 26.879 | 75.349 | 72.848 |  | *Pf* | *Pf* |  | *Pf* |  |
| 172 | 33.071 | 75.368 | 72.845 | 79.640 | *Pf* | *Pf* |  | *Pf* |  |
| 173 | 27.358 | 75.368 | 72.845 |  | *Pf* | *Pf* |  | *Pf* |  |
| 174 | 22.764 | 75.338 | 72.840 |  | *Pf* | *Pf* |  | *Pf* |  |
| 175 | 34.857 | 75.362 | 72.839 |  | *Pf* | *Pf* |  | *Pf* |  |
| 176 | 25.000 | 75.361 | 72.838 |  | *Pf* | *Pf* |  | *Pf* |  |
| 177 | 26.724 | 75.347 | 72.835 |  | *Pf* | *Pf* |  | *Pf* |  |
| 178 | 30.574 | 75.344 | 72.832 |  | *Pf* | *Pf* |  | *Pf* |  |
| 179 | 30.453 | 75.306 | 72.831 |  | *Pf* | *Pf* |  | *Pf* |  |
| 180 | 27.700 | 75.302 | 72.827 |  | *Pf* | *Pf* |  | *Pf* |  |
| 181 | 32.011 | 75.343 | 72.826 |  | *Pf* | *Pf* |  | *Pf* |  |
| 182 | 26.873 | 75.342 | 72.825 |  | *Pf* | *Pf* |  | *Pf* |  |
| 183 | 33.115 | 75.300 | 72.824 |  | *Pf* | *Pf* |  | Negative |  |
| 184 | 33.006 | 75.324 | 72.823 | 80.175 | *Pf* | *Pf* |  | Negative |  |
| 185 | 24.000 | 75.323 | 72.822 |  | *Pf* | *Pf* |  | *Pf* |  |
| 186 | 23.656 | 75.315 | 72.817 |  | *Pf* | *Pf* |  | *Pf* |  |
| 187 | 26.215 | 75.352 | 72.814 |  | *Pf* | *Pf* |  | *Pf* |  |
| 188 | 27.138 | 75.325 | 72.813 |  | *Pf* | *Pf* |  | *Pf* |  |
| 189 | 30.124 | 75.310 | 72.812 |  | *Pf* | *Pf* |  | *Pf* |  |
| 190 | 22.207 | 75.327 | 72.802 |  | *Pf* | *Pf* |  | *Pf* |  |
| 191 | 30.711 | 75.324 | 72.798 |  | *Pf* | *Pf* |  | *Pf* |  |
| 192 | 18.268 | 75.298 | 72.797 |  | *Pf* | *Pf* |  | *Pf* |  |
| 193 | 36.360 | 75.248 | 72.787 |  | *Pf* | *Pf* |  | Negative |  |
| 194 | 23.362 | 75.323 | 72.786 |  | *Pf* | *Pf* |  | *Pf* |  |
| 195 | 29.406 | 75.271 | 72.784 |  | *Pf* | *Pf* |  | *Pf* |  |
| 196 | 26.194 | 75.295 | 72.783 |  | *Pf* | *Pf* |  | *Pf* |  |
| 197 | 28.205 | 75.276 | 72.776 |  | *Pf* | *Pf* |  | *Pf* |  |
| 198 | 21.370 | 75.251 | 72.775 |  | *Pf* | *Pf* |  | Negative |  |
| 199 | 28.994 | 75.299 | 72.773 |  | *Pf* | *Pf* |  | Negative |  |
| 200 | 24.819 | 75.323 | 72.773 |  | *Pf* | *Pf* |  | *Pf* |  |
| 201 | 26.914 | 75.273 | 72.772 |  | *Pf* | *Pf* |  | *Pf* |  |
| 202 | 25.894 | 75.286 | 72.763 |  | *Pf* | *Pf* |  | *Pf* |  |
| 203 | 29.494 | 75.274 | 72.762 |  | *Pf* | *Pf* |  | *Pf* |  |
| 204 | 25.645 | 75.250 | 72.749 |  | *Pf* | *Pf* |  | *Pf* |  |
| 205 | 20.863 | 75.249 | 72.748 |  | *Pf* | *Pf* |  | *Pf* |  |
| 206 | 24.299 | 75.268 | 72.727 |  | *Pf* | *Pf* |  | *Pf* |  |
| 207 | 31.945 | 75.201 | 72.725 |  | *Pf* | *Pf* |  | *Pf* |  |
| 208 | 31.530 | 75.249 | 72.723 |  | *Pf* | *Pf* |  | *Pf* |  |
| 209 | 29.470 | 75.248 | 72.722 |  | *Pf* | *Pf* |  | *Pf* |  |
| 210 | 27.821 | 75.194 | 72.693 |  | *Pf* | *Pf* |  | *Pf* |  |
| 211 | 26.459 | 75.221 | 72.684 |  | *Pf* | *Pf* |  | *Pf* |  |
| 212 | 26.560 | 75.300 | 72.625 |  | *Pf* | *Pf* |  | *Pf* |  |
| 213 | 33.369 | 74.892 | 72.405 |  | *Pf* | *Pf* |  | *Pf* |  |
| 214 | 22.999 | 75.507 | 72.984 |  | *Pf* | *Pf* |  | *Pf* |  |
| 215 | 23.575 | 75.432 | 72.934 |  | *Pf* | *Pf* |  | *Pf* |  |
| 216 | 35.985 | 75.451 | 72.888 |  | *Pf* | *Pf* |  | No result |  |
| 217 | 34.674 | 75.382 | 72.859 |  | *Pf* | *Pf* |  | *Pf* |  |
| 218 | Undetermined | 75.332 | 72.809 |  | *Pf* | *Pf* |  | *Pf* |  |
| 219 | 30.660 | 72.937 | 75.310 | 70.389 | *Pf* | *Pf* |  | *Pf* |  |
| 220 | 21.413 | 72.826 | 70.210 | 75.168 | *Pf* | *Pf* |  | *Pf* |  |
| 221 | 34.784 | 74.777 | 72.327 |  | *Pv* | *Pf* |  | *Pf* |  |
| 222 | Undetermined | 88.554 | 79.528 | 70.251 | *Negative* | *Pf* |  | *Pf* |  |
| 223 | 23.074 | 72.975 | 75.500 | 74.775 | *Pf* | *Pf* |  | *Pf* |  |
| 224 | 32.986 | 72.953 | 75.478 |  | *Pf* | *Pf* |  | *Pf* |  |
| 225 | 28.783 | 75.372 | 72.911 |  | *Pf* | *Pf* |  | *Pf* |  |
| 226 | 27.892 | 75.324 | 72.837 |  | *Pf* | *Pf* |  | *Pf* |  |
| 227 | 30.131 | 75.335 | 72.862 |  | *Pf* | *Pf* |  | *Pf* |  |
| 228 | 29.780 | 75.540 | 73.042 | 79.188 | *Pf* | *Pf* |  | *Pf* |  |
| 229 | 28.944 | 75.364 | 72.866 |  | *Pf* | *Pf* |  | *Pf* |  |
| 230 | 28.235 | 75.352 | 72.840 |  | *Pf* | *Pf* |  | *Pf* |  |
| 231 | 27.484 | 75.289 | 72.791 |  | *Pf* | *Pf* |  | *Pf* |  |
| 232 | 27.873 | 75.255 | 72.755 |  | *Pf* | *Pf* |  | *Pf* |  |
| 233 | 21.957 | 75.349 | 72.837 |  | *Pf* | *Pf* |  | *Pf* |  |
| 234 | 22.633 | 75.453 | 72.903 |  | *Pf* | *Pf* |  | *P spp* |  |
| 235 | 29.653 | 72.953 | 75.103 |  | *Pf* | *Pf* |  | No result |  |
| 236 | 26.039 | 75.553 | 73.053 |  | *Pf* | *Pf* |  | No result |  |
| 237 | 25.749 | 75.375 | 72.850 |  | *Pf* | *Pf* |  | No result |  |
| 238 | 23.427 | 75.339 | 72.841 |  | *Pf* | *Pf* |  | No result |  |
| 239 | 23.126 | 75.332 | 72.832 |  | *Pf* | *Pf* |  | No result |  |
| 240 | 18.307 | 75.343 | 72.795 |  | *Pf* | *Pf* |  | No result |  |
| 241 | 23.846 | 75.196 | 72.684 |  | *Pf* | *Pf* |  | No result |  |
| 242 | 30.352 | 75.360 | 72.887 | 79.657 | *Pf* | *Pf Po* |  | *Pf* |  |
| 243 | 14.697 | 75.348 | 72.798 |  | *Pf* | *Pf Po* | C | *Pf* |  |
| 244 | 23.218 | 75.399 | 72.913 |  | *Pf* | *Pf Po* |  | *Pf Po* |  |
| 245 | 25.169 | 73.867 | 75.591 | 70.794 | *Pf Poc* | *Pf Po* | C | *Po* |  |
| 246 | Undetermined | 92.808 | 65.429 | 67.129 | *Negative* | *Pm* |  | *Pm* |  |
| 247 | 28.784 | 73.550 |  |  | *Pm* | *Pm* |  | *Pm* |  |
| 248 | 28.911 | 73.937 |  |  | *Pm* | *Pm* |  | *Pm* |  |
| 249 | 24.777 | 74.079 |  |  | *Pm* | *Pm* |  | *Pm* |  |
| 250 | 26.088 | 73.803 |  |  | *Pm* | *Pm* |  | *Pm* |  |
| 251 | 26.933 | 74.200 |  |  | *Pm* | *Pm* |  | *Pm* |  |
| 252 | 26.600 | 73.875 |  |  | *Pm* | *Pm* |  | *Pm* |  |
| 253 | 28.110 | 73.926 |  |  | *Pm* | *Pm* |  | *Pm* |  |
| 254 | 29.451 | 74.052 |  |  | *Pm* | *Pm* |  | *P spp* |  |
| 255 | 26.636 | 73.949 |  |  | *Pm* | *Pm* |  | *Pm* |  |
| 256 | 27.817 | 73.925 |  |  | *Pm* | *Pm* |  | *Pm* |  |
| 257 | 21.247 | 73.799 |  |  | *Pm* | *Pm* |  | *Pm* |  |
| 258 | 22.080 | 73.924 |  |  | *Pm* | *Pm* |  | *Pm* |  |
| 259 | 25.140 | 73.923 |  |  | *Pm* | *Pm* |  | *Pm* |  |
| 260 | 30.493 | 74.048 |  |  | *Pm* | *Pm* |  | *Pm* |  |
| 261 | 29.667 | 73.724 |  |  | *Pm* | *Pm* |  | *Pm* |  |
| 262 | 21.891 | 73.899 |  |  | *Pm* | *Pm* |  | *Pm* |  |
| 263 | 25.987 | 74.551 |  |  | *Pv* | *Pm* |  | *Pm* |  |
| 264 | 24.245 | 74.497 |  |  | *Pv* | *Pm* |  | *Pm* |  |
| 265 | Undetermined | 60.648 | 93.428 | 78.801 | *Negative* | *Pm* |  | *Pm* |  |
| 266 | 25.908 | 73.789 | 70.641 |  | *Poc* | *Pm Po* |  | *Pm* |  |
| 267 | 25.755 | 74.312 |  |  | *Pm* | *Po* | C | *Po* |  |
| 268 | 27.268 | 74.325 |  |  | *Pm* | *Po* | C | *Po* |  |
| 269 | 30.076 | 73.876 | 70.628 |  | *Poc* | *Po* | C | *Po* | *Benin* |
| 270 | 25.000 | 73.837 | 69.615 |  | *Poc* | *Po* | C | *Po* | *Guinea* |
| 271 | 28.734 | 73.376 | 70.175 |  | *Poc* | *Po* | C | *Po* | Cameroon |
| 272 | 26.550 | 73.571 | 70.349 |  | *Poc* | *Po* | C | *Po* | *Guinea* |
| 273 | 22.966 | 73.600 | 70.374 |  | *Poc* | *Po* | C | *Po* | *Ivory Coast* |
| 274 | 28.320 | 73.638 | 70.391 |  | *Poc* | *Po* | C | *Po* | *Central African Republic* |
| 275 | 25.238 | 73.693 | 70.446 |  | *Poc* | *Po* | C | *Po* | *Ivory Coast* |
| 276 | 21.653 | 73.639 | 70.466 |  | *Poc* | *Po* | C | *Po* | *Burkina Faso* |
| 277 | 25.265 | 73.711 | 70.488 |  | *Poc* | *Po* | C | *Po* | *Nigeria* |
| 278 | 29.626 | 73.750 | 70.500 |  | *Poc* | *Po* | C | *Po* | *Central African Republic* |
| 279 | 25.000 | 73.711 | 70.513 |  | *Poc* | *Po* | C | *Po* | *Central African Republic* |
| 280 | 26.361 | 73.750 | 70.525 |  | *Poc* | *Po* | C | *Po* | *Guinea* |
| 281 | 29.875 | 73.801 | 70.550 |  | *Poc* | *Po* | C | *Po* | *Ivory Coast* |
| 282 | 25.235 | 73.752 | 70.551 |  | *Poc* | *Po* | C | *Po* | *Central African Republic* |
| 283 | 28.983 | 73.616 | 70.144 |  | *Poc* | *Po* | C | *Po* | *Benin* |
| 284 | 26.202 | 73.799 | 70.574 |  | *Poc* | *Po* | C | *Po* | *Ivory Coast* |
| 285 | 30.747 | 73.680 | 70.433 |  | *Poc* | *Po* | C | *Po* | *Ivory Coast* |
| 286 | 26.869 | 73.699 | 70.477 |  | *Poc* | *Po* | C | *Po* | *Congo* |
| 287 | 22.398 | 73.746 | 70.532 |  | *Poc* | *Po* | C | *Po* | *Ivory Coast* |
| 288 | 26.442 | 73.747 | 70.647 |  | *Poc* | *Po* | C | *Po* | *Ivory Coast* |
| 289 | 28.150 | 73.749 | 70.501 |  | *Poc* | *Po* | C | *Po* | *Ivory Coast* |
| 290 | 26.358 | 73.751 | 70.626 |  | *Poc* | *Po* | C | *Po* | *Cameroon* |
| 291 | 28.024 | 73.749 | 70.552 |  | *Poc* | *Po* | C | *Po* | *Benin* |
| 292 | 30.481 | 73.750 | 70.553 |  | *Poc* | *Po* | C | *Po* | *Ivory Coast* |
| 293 | 31.896 | 73.774 | 70.552 |  | *Poc* | *Po* | C | *Po* | *Central African Republic* |
| 294 | 26.829 | 73.777 | 70.503 |  | *Poc* | *Po* | C | *Po* | *Mali* |
| 295 | 26.806 | 73.799 | 70.576 |  | *Poc* | *Po* | C | *Po* | *Ivory Coast* |
| 296 | 18.798 | 73.823 | 70.623 |  | *Poc* | *Po* | C | *Po* | *Senegal* |
| 297 | 30.212 | 73.850 | 70.674 |  | *Poc* | *Po* | C | *Po* | *Nigeria* |
| 298 | 25.957 | 73.888 | 70.690 |  | *Poc* | *Po* | C | *Po* | *Ivory Coast* |
| 299 | 26.273 | 74.113 | 70.715 |  | *Poc* | *Po* | C | *Po* | *Ivory Coast* |
| 300 | 25.340 | 73.764 | 70.716 |  | *Poc* | *Po* | C | *Po* | *Central African Republic* |
| 301 | 22.724 | 73.881 | 70.684 |  | *Poc* | *Po* | C | *Po* | *Ivory Coast* |
| 302 | 29.633 | 73.749 | 70.501 |  | *Poc* | *Po* | C | *Po* | *Ivory Coast* |
| 303 | 27.203 | 73.040 | 70.756 |  | *Pow* | *Po* | W | *Po* | *Ivory Coast* |
| 304 | 28.440 | 73.192 | 71.010 |  | *Pow* | *Po* | W | *Po* | *Central African Republic* |
| 305 | 25.000 | 73.247 | 70.897 |  | *Pow* | *Po* | W | *Po* | *Congo* |
| 306 | 28.417 | 73.401 | 71.026 |  | *Pow* | *Po* | W | *Po* | *Ghana* |
| 307 | 26.029 | 73.335 | 71.036 |  | *Pow* | *Po* | W | *Po* | *Ivory Coast* |
| 308 | 23.367 | 73.317 |  |  | *Pow* | *Po* | W | *Po* | *Ivory Coast* |
| 309 | 29.127 | 73.324 | 71.449 |  | *Pow* | *Po* | W | *Po* | *Cameroon* |
| 310 | 23.087 | 73.369 | 71.085 |  | *Pow* | *Po* | W | *Po* | *Central African Republic* |
| 311 | 26.513 | 73.373 | 71.048 |  | *Pow* | *Po* | W | *Po* | *Cameroon* |
| 312 | 30.554 | 73.445 | 71.187 |  | *Pow* | *Po* | W | *Po* | *Ivory Coast* |
| 313 | 25.294 | 73.366 | 71.067 |  | *Pow* | *Po* | W | *Po* | *Cameroon* |
| 314 | 18.000 | 73.449 | 71.107 |  | *Pow* | *Po* | W | *Po* | *Ivory Coast* |
| 315 | 23.000 | 73.485 | 71.087 |  | *Pow* | *Po* | W | *Po* | *Cameroon* |
| 316 | 24.174 | 73.365 | 71.091 |  | *Pow* | *Po* | W | *Po* | *Cameroon* |
| 317 | 29.046 | 73.401 | 71.101 |  | *Pow* | *Po* | W | *Po* | *Ivory Coast* |
| 318 | 21.560 | 73.468 | 71.058 |  | *Pow* | *Po* | W | *Po* | *Cameroon* |
| 319 | 27.168 | 73.474 | 71.199 |  | *Pow* | *Po* | W | *Po* | *Ivory Coast* |
| 320 | 27.783 | 73.457 | 71.134 |  | *Pow* | *Po* | W | *Po* | *Sierra Leone* |
| 321 | 31.753 | 73.477 | 71.152 |  | *Pow* | *Po* | W | *P spp* | *Togo* |
| 322 | 27.000 | 73.463 | 71.165 |  | *Pow* | *Po* | W | *Po* | *Ivory Coast* |
| 323 | 26.663 | 73.482 | 71.182 |  | *Pow* | *Po* | W | *Po* | *Cameroon* |
| 324 | 32.717 | 73.416 | 71.167 |  | *Pow* | *Po* | W | *Po* | *No information* |
| 325 | 22.368 | 73.518 | 71.170 |  | *Pow* | *Po* | W | *Po* | *No information* |
| 326 | 25.000 | 73.539 | 71.216 |  | *Pow* | *Po* | W | *Po* | *Cameroon* |
| 327 | 27.278 | 73.278 | 71.328 |  | *Pow* | *Po* | W | *Po* | *Ivory Coast* |
| 328 | 24.360 | 73.296 | 71.347 |  | *Pow* | *Po* | W | *Po* | *Ivory Coast* |
| 329 | 27.773 | 73.358 | 71.383 |  | *Pow* | *Po* | W | *Po* | *Mali* |
| 330 | 27.638 | 73.357 | 71.432 |  | *Pow* | *Po* | W | *Po* | *Congo* |
| 331 | 23.850 | 73.512 | 71.539 |  | *Pow* | *Po* | W | *Po* | *Cameroon* |
| 332 | 28.645 | 73.270 | 71.321 |  | *Pow* | *Po* | W | *Po* | *Non information* |
| 333 | 31.786 | 73.679 | 71.329 |  | *Pow* | *Po* | W | *Po* | *Ivory Coast* |
| 334 | 22.956 | 73.839 |  |  | *Pm* | *Po* | W | *Po* |  |
| 335 | 22.319 | 73.242 | 71.288 |  | *Pow* | *Po* | W | *Po* | *Ivory Coast* |
| 336 | 26.000 | 74.112 |  |  | *Pm* | *Pm Po* | C | *Po* |  |
| 337 | 23.832 | 75.248 |  |  | *Pf* | *Pv* |  | *Pv* |  |
| 338 | 26.858 | 74.835 |  |  | *Pv* | *Pv* |  | *Pv* |  |
| 339 | 26.732 | 74.737 |  |  | *Pv* | *Pv* |  | *Pv* |  |
| 340 | 21.154 | 74.699 |  |  | *Pv* | *Pv* |  | *Pv* |  |
| 341 | 22.310 | 74.798 |  |  | *Pv* | *Pv* |  | *Pv* |  |
| 342 | 34.463 | 74.811 |  |  | *Pv* | *Pv* |  | *Pv* |  |
| 343 | 22.439 | 74.916 |  |  | *Pv* | *Pv* |  | *Pv* |  |
| 344 | 25.418 | 74.783 |  |  | *Pv* | *Pv* |  | *Pv* |  |
| 345 | 23.203 | 74.790 |  |  | *Pv* | *Pv* |  | *Pv* |  |
| 346 | 20.075 | 74.868 |  |  | *Pv* | *Pv* |  | *Pv* |  |
| 347 | 25.065 | 74.721 |  |  | *Pv* | *Pv* |  | *Pv* |  |
| 348 | 23.703 | 74.721 |  |  | *Pv* | *Pv* |  | *Pv* |  |
| 349 | 22.872 | 74.723 |  |  | *Pv* | *Pv* |  | *Pv* |  |
| 350 | 27.676 | 74.882 |  |  | *Pv* | *Pv* |  | *Pv* |  |
| 351 | 21.251 | 74.871 |  |  | *Pv* | *Pv* |  | *Pv* |  |
| 352 | 22.000 | 74.920 |  |  | *Pv* | *Pv* |  | *Pv* |  |
| 353 | 25.766 | 74.670 |  |  | *Pv* | *Pv* |  | *Pv* |  |
| 354 | 24.280 | 72.990 | 75.513 |  | *Pf* | *Pf* |  | *Pf* |  |
| 355 | 22.302 | 72.888 | 75.386 |  | *Pf* | *Pf* |  | *Pf* |  |
| 356 | 24.370 | 75.437 | 72.914 |  | *Pf* | *Pf* |  | *Pf* |  |

Supplementary Table S1 – qPCR-HRM, qPCR-Taqman, microscopy and nested PCR result for each sample.

A)


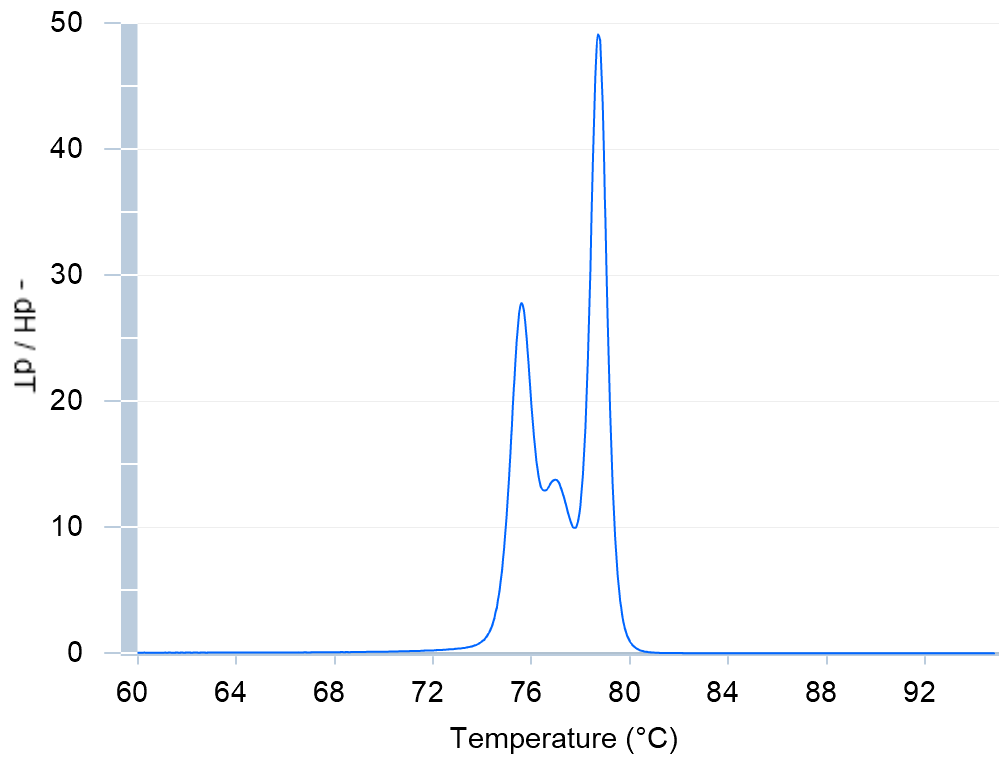


Derivative melt curved obtained with uMELT BATCH2.0 for the 4 genomic sequences coding for *Plasmodium falciparum* 18S RNA subunit. Blake and Delcourt (Nucleic Acids, 1998) thermodynamic library was used, with a temperature range from 60 to 95°C. Resolution setting was very high. The profile curve displays 2 Tm, respectively **75.6°C** and **78.7°C**, which results from the slight differences in the genomic sequences.


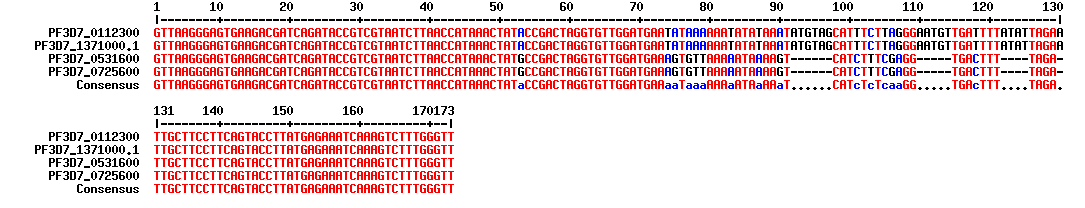


18S RNA sequences from *Plasmodium falciparum* 3D7 alignement using Multialin (Corpet Nucl. Acids Res 1998). 4 chromosome encode *Plasmodium falciparum* 18S RNA, however, sequences from chromosome 1 and 13 (PF3D7_0112300 and PF3D7_1371000.1) are similar, as well as sequences from chromosome 5 and 7.

B)


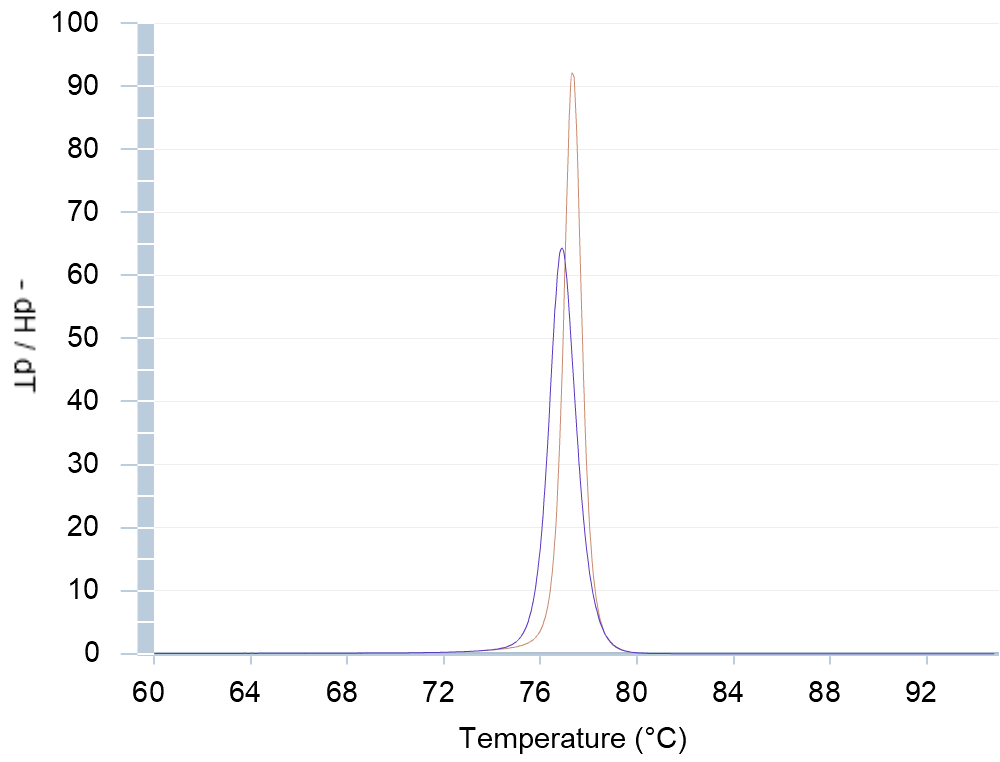


Derivative melt curved obtained with uMELT BATCH2.0 for the genomic sequences coding for *Plasmodium wallikeri* and *Plasmodium curtisi* 18S RNA gene.

**Predictive Tm for *Plasmodium ovale wallikeri* = 77,4°C**

**Predictive Tm for *Plasmodium ovale curtisi* = 76,8°C**

C)


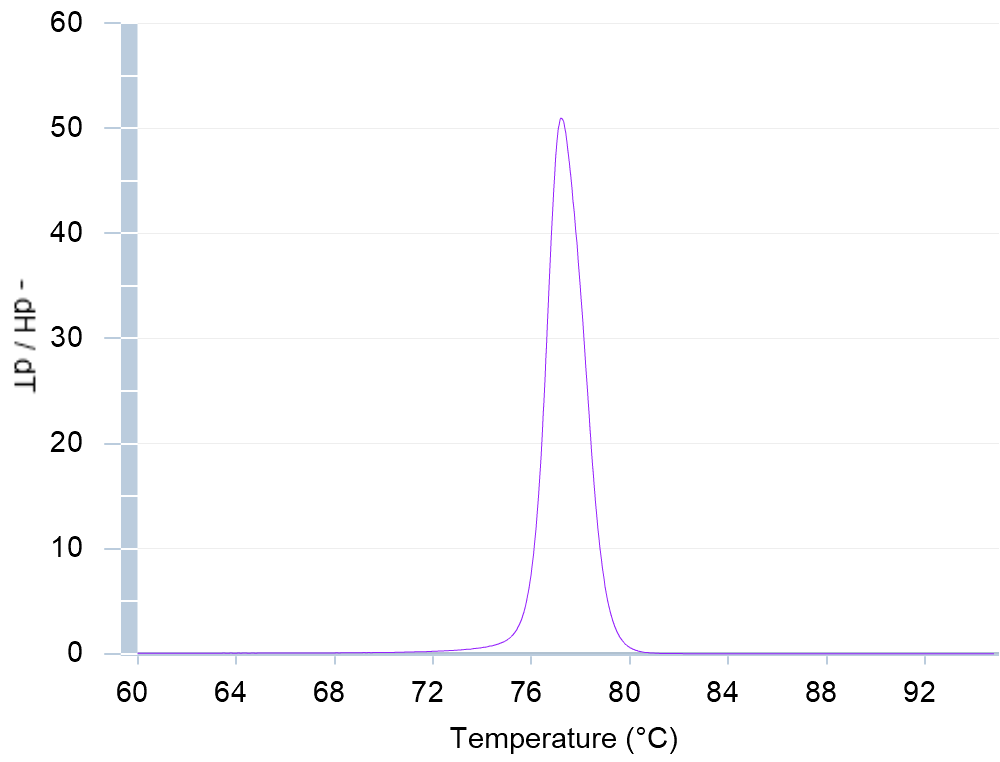


Derivative melt curved obtained with uMELT BATCH2.0 for the genomic sequences coding for *Plasmodium malariae* 18S RNA gene.

**Predictive Tm =77,2°C**

D)


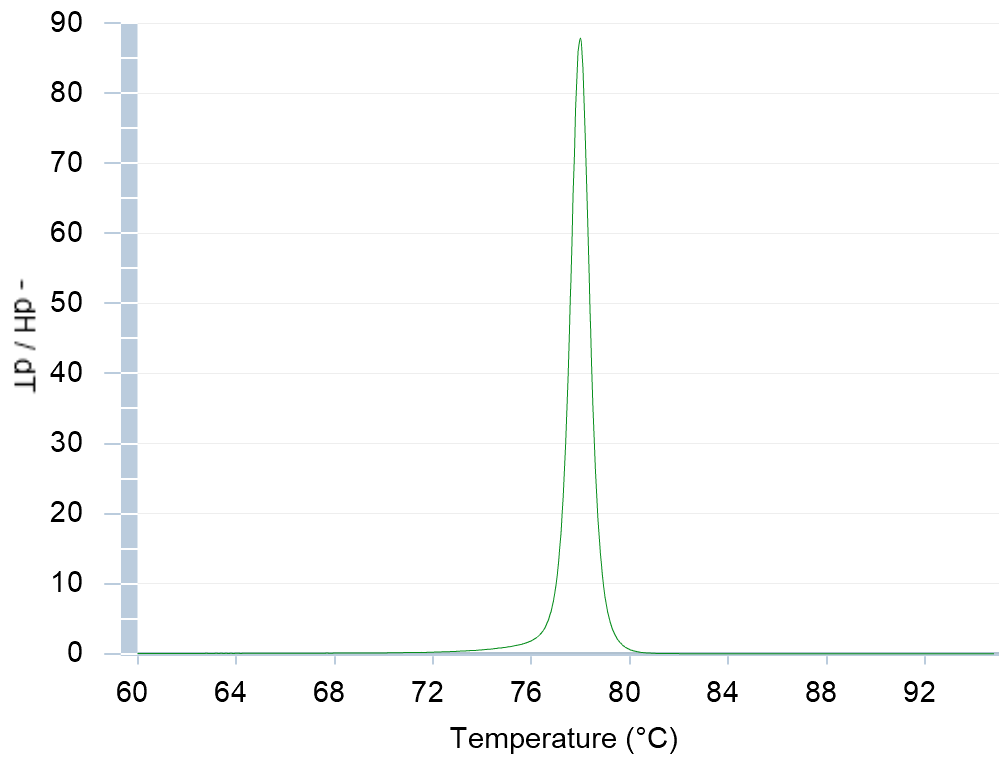


Derivative melt curved obtained with uMELT BATCH2.0 for the genomic sequences coding for *Plasmodium vivax* 18S RNA gene.

**Predictive Tm = 77,9°C**

Supplementary Figure 2 – Derivate melt curved obtained with uMELT Batch 2.0. A: *Plasmodium falciparum*, B: *Plasmodium ovale wallikeri* and *Plasmodium ovale curtisi*, C: *Plasmodium malariae*, D: *Plasmodium vivax*


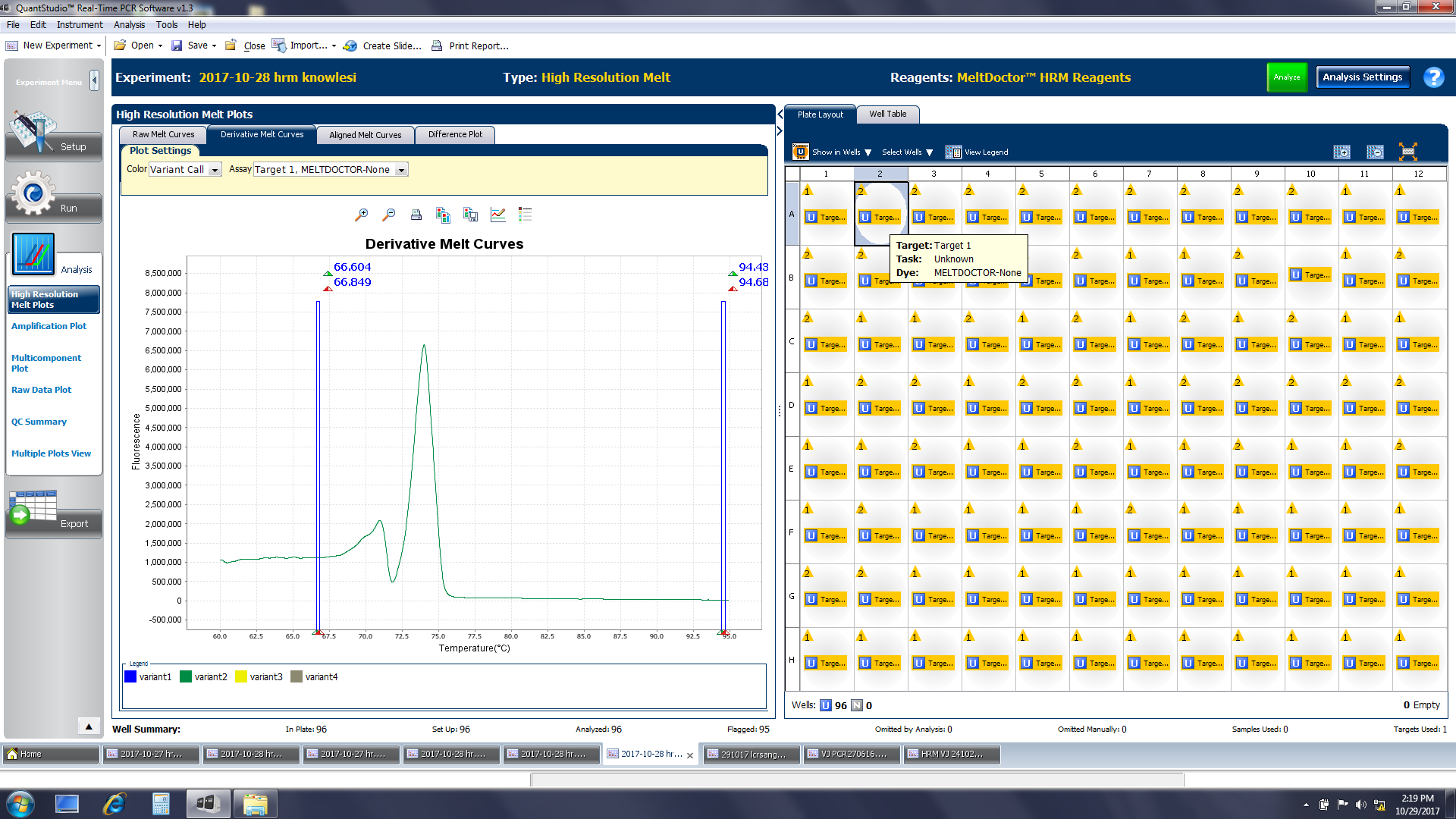


**Poc9-Pow1**

*Pow* Tm1

*Poc* Tm 1 and Tm2


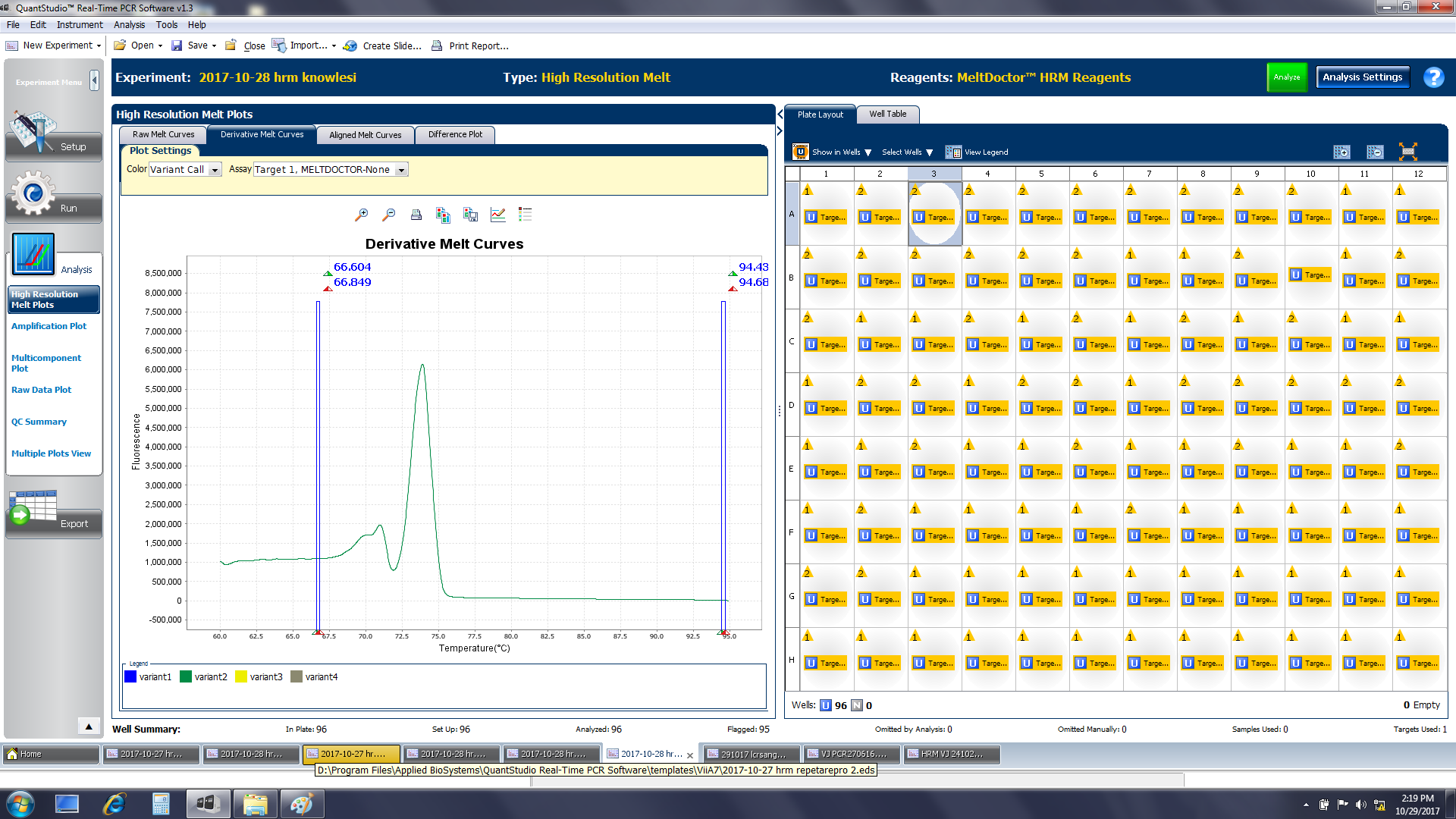


**Poc8-Pow2**


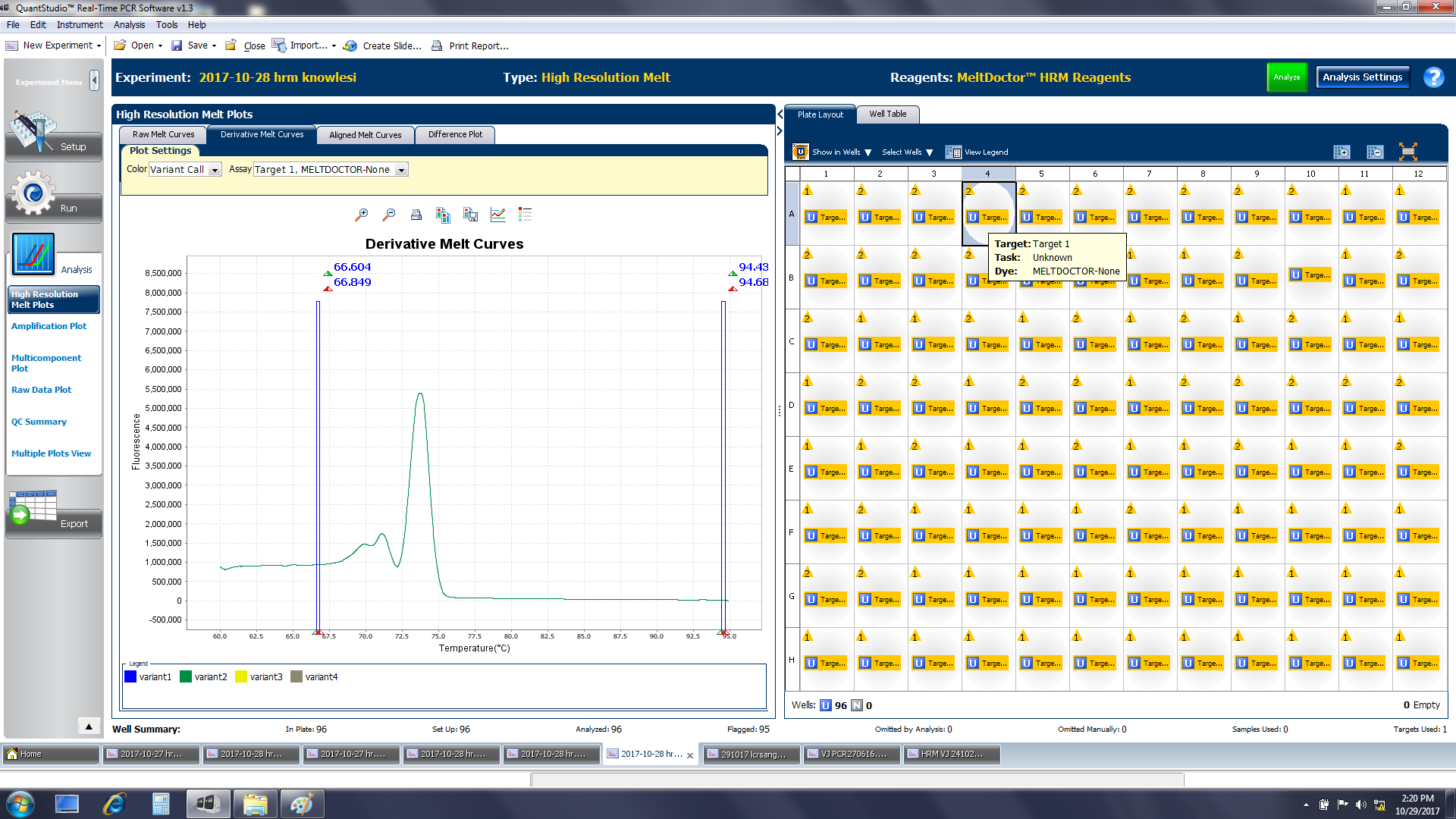


**Poc7-Pow3**


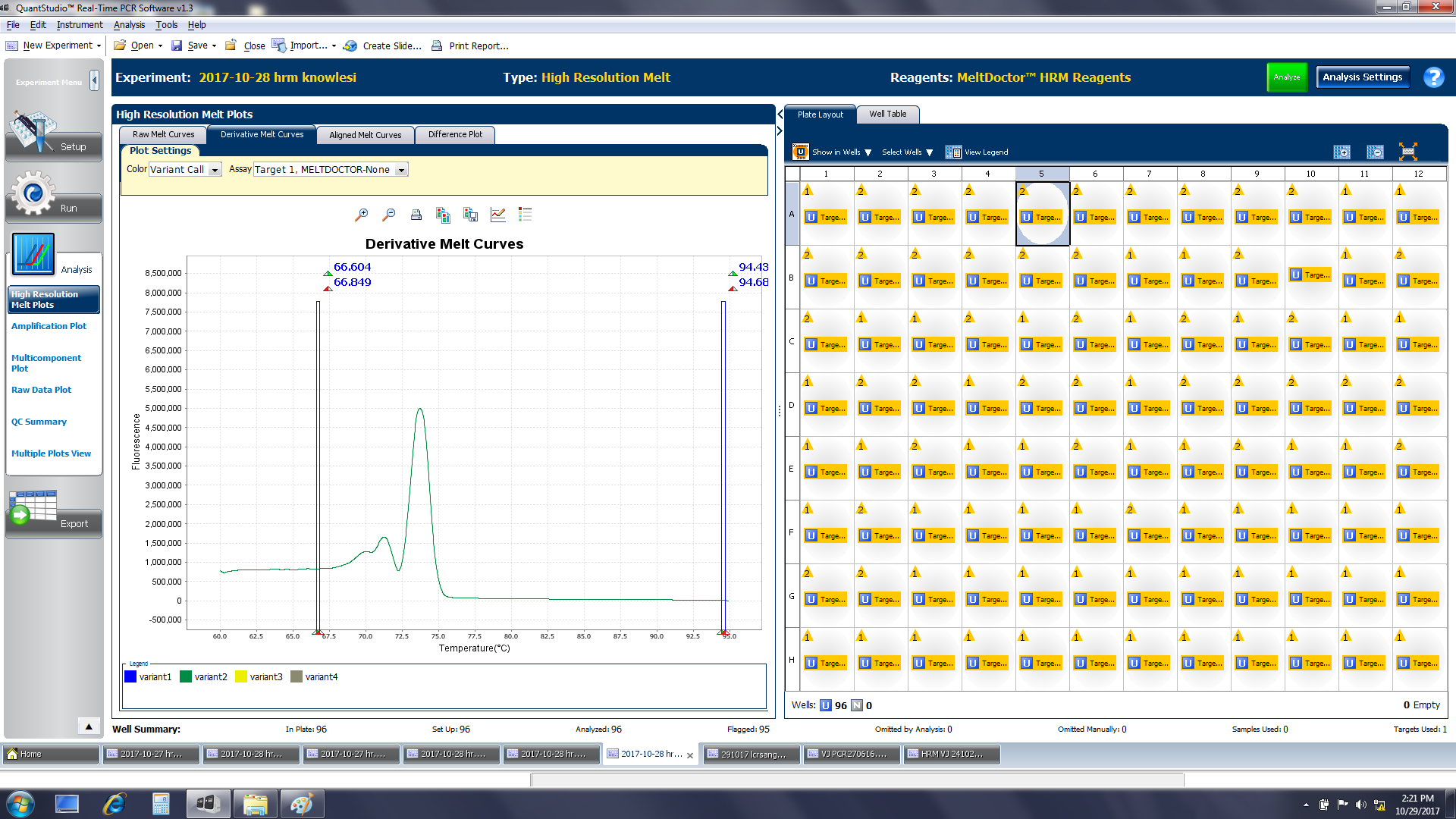


**Poc6-Pow4**


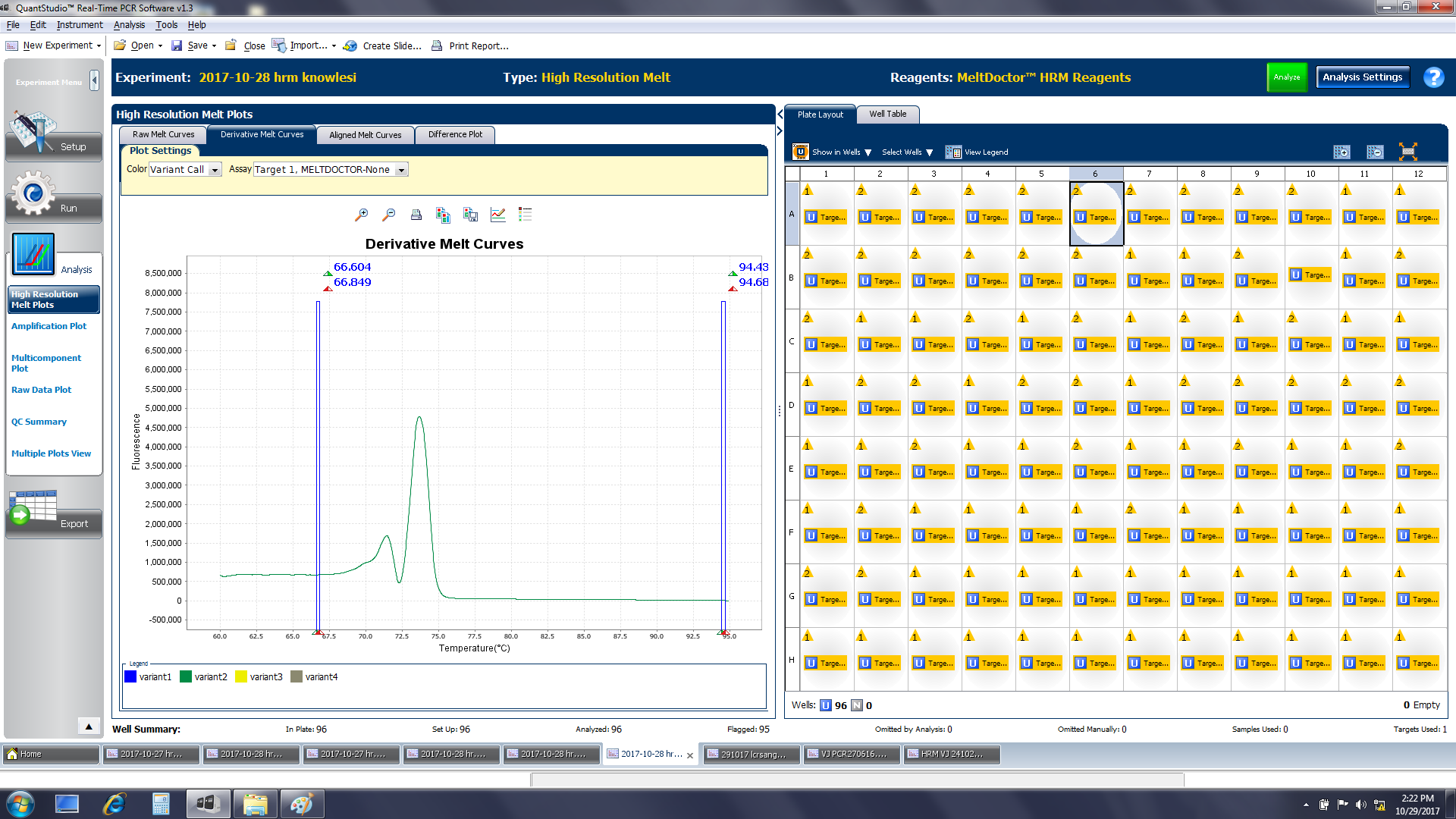


**Poc5-Pow5**


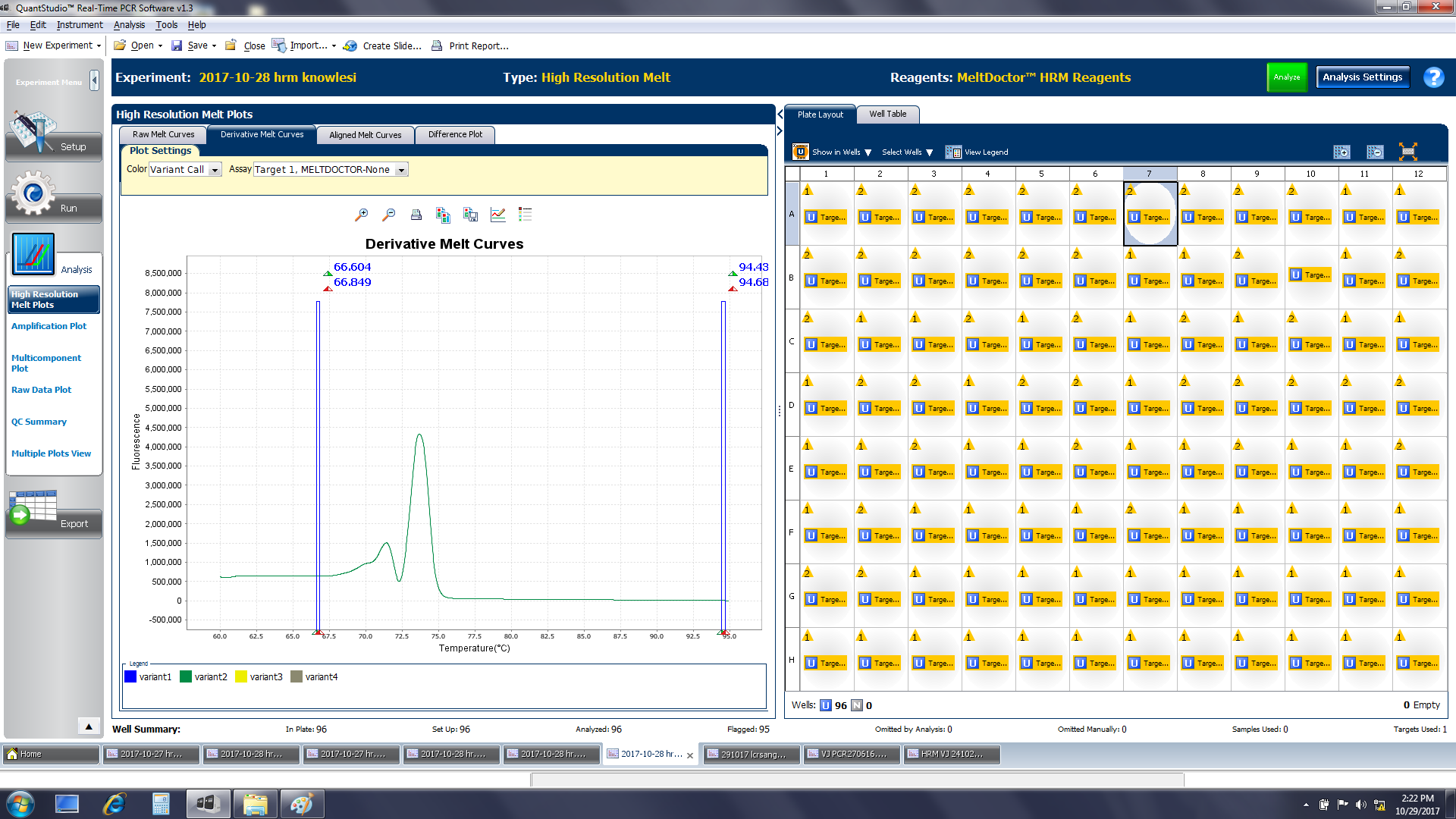


**Poc4-Pow6**


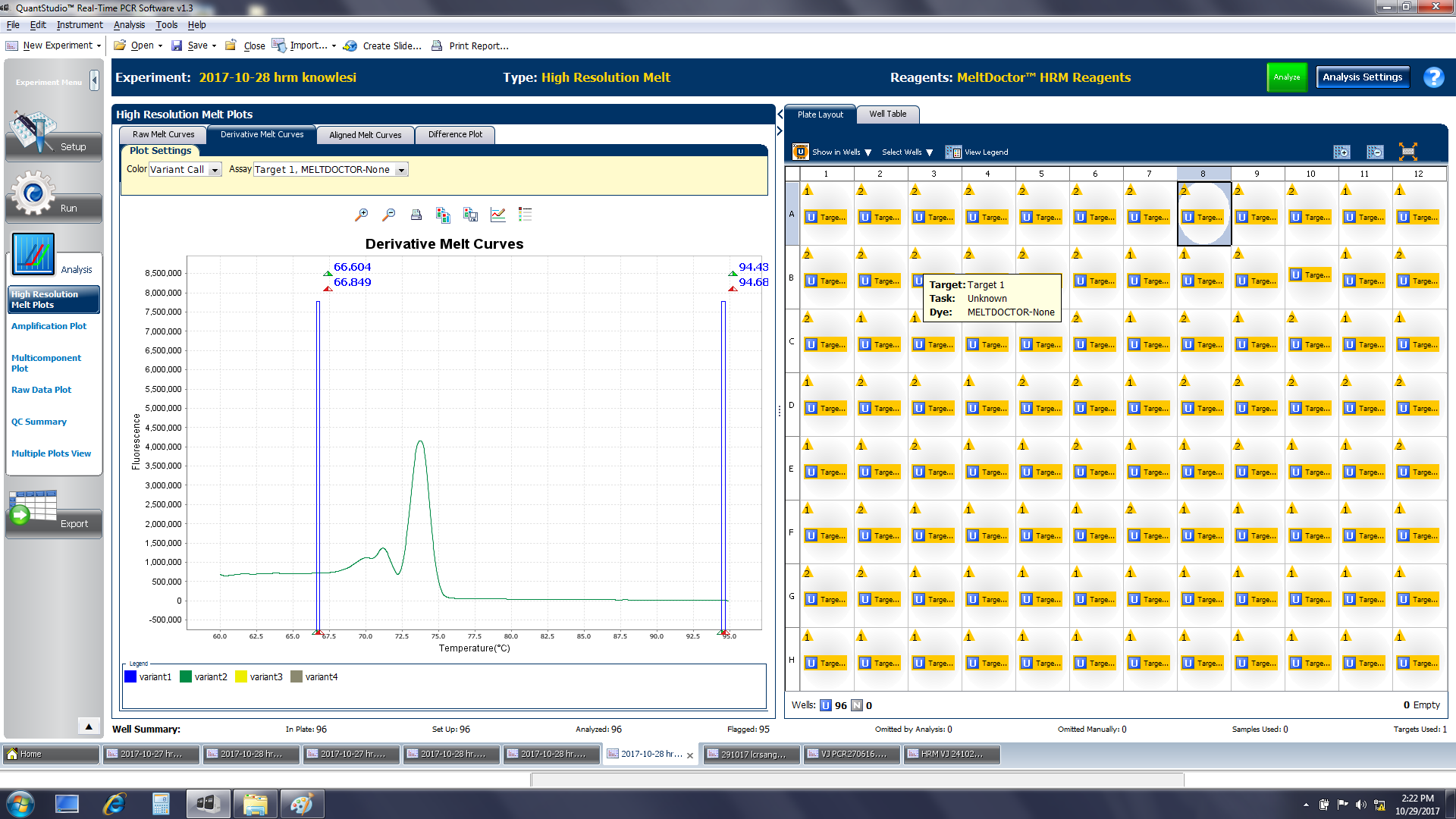


**Poc3-Pow7**


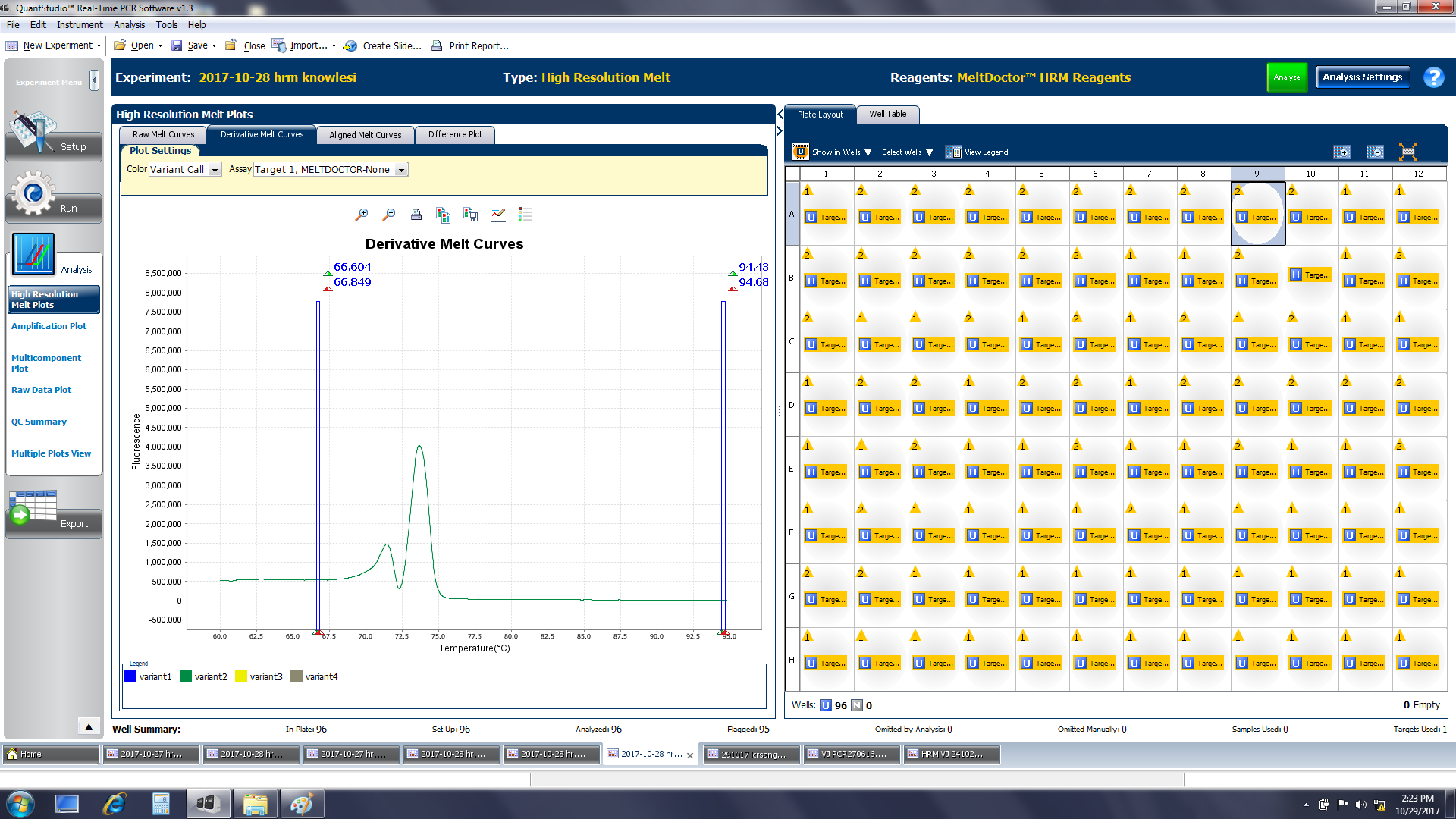


**Poc2-Pow8**


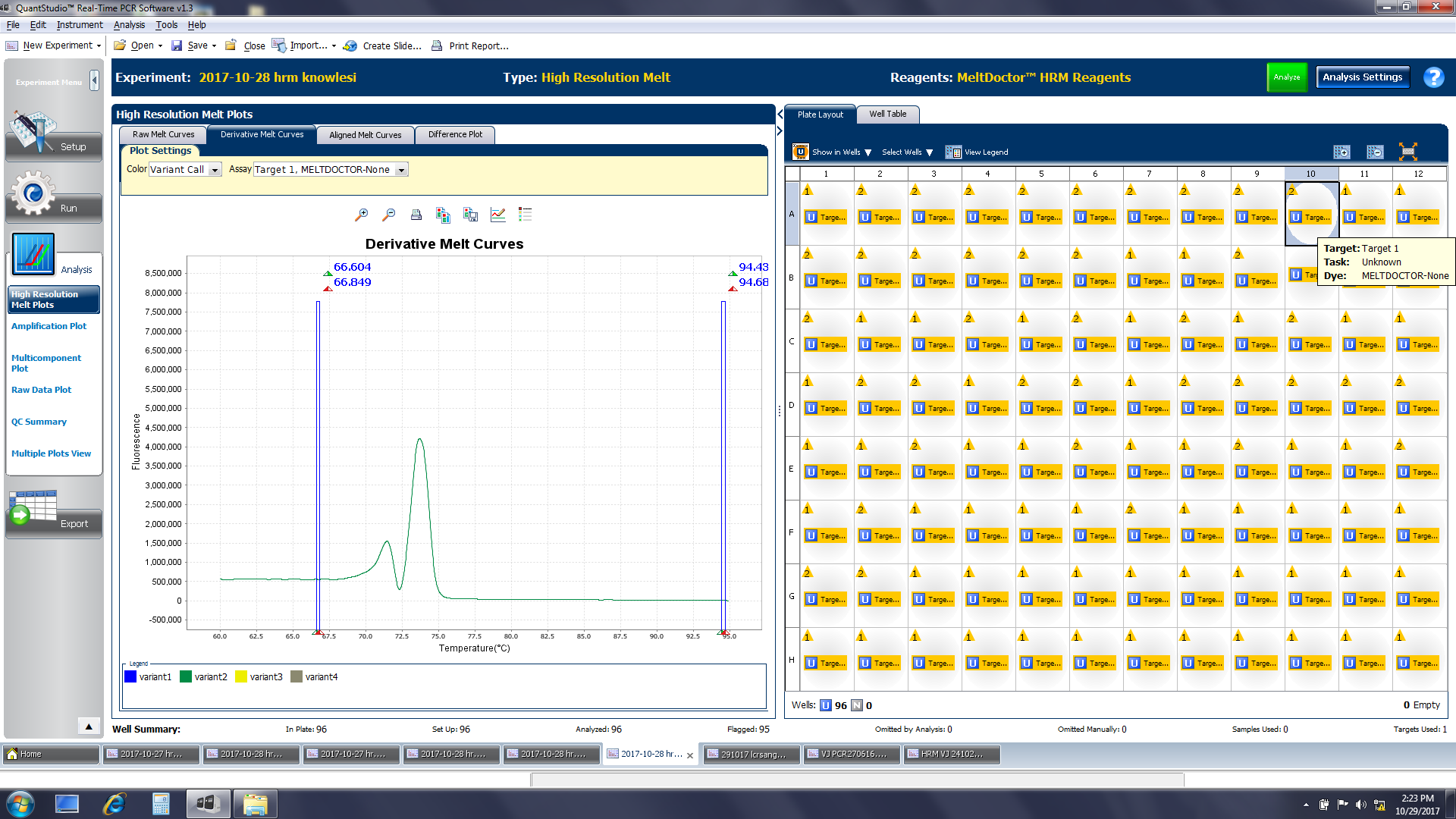


**Poc1-Pow9**

Supplementary Figure S3 - Derivated melt curved obtained for the artificial mixes from *Plasmodium ovale curtisi* and *Plasmodium ovale wallikeri.* We prepared 9 artificial samples with various ratio of *Poc* and *Pow* (9/1 ; 8/2 ; 7/3 ; 6/4 ; 5/5 ; for both). Initial parasite density of *Poc* was 3000 p/µL and initial parasite density of *Pow* was 6000 p/µL. Co-identification was possible for all mixed samples except when *Pow* was the majority species (8/2 and 9/1 ratio).

|  | Ct | Tm 1 | Tm 2 | Tm 3 |
| --- | --- | --- | --- | --- |
| *Toxoplasma gondii* | 33,420 | 76,570 |  |  |
| *Toxoplasma gondii* | 33,598 | 76,519 |  |  |
| *Toxoplasma gondii* | 33,738 | 76,519 |  |  |
| *Leishmania sp* | Undetermined |  |  |  |
| *Leishmania sp* | Undetermined |  |  |  |
| *Leishmania sp* | Undetermined |  |  |  |
| *Babesia sp* | Undetermined |  |  |  |
| *Wuchereria bancrofti* | 37,567 | 76,943 | 80,278 |  |
| *Wuchereria bancrofti* | 36,940 | 69,975 | 77,690 | 80,303 |
| *Wuchereria bancrofti* | 37,056 | 69,999 | 77,665 | 80,328 |
| *Onchocerca volvulus* | Undetermined |  |  |  |
| *Onchocerca volvulus* | Undetermined |  |  |  |
| *Onchocerca volvulus* | Undetermined |  |  |  |
| *Loa loa* | Undetermined |  |  |  |
| *Loa loa* | Undetermined |  |  |  |

Supplementary Table S4 – Ct and Tm for specificity test

We performed the qPCR-HRM on several samples positive for other parasite’s than *Plasmodium*. No amplification was observed for *Leishmania sp* positive samples, *Babesia sp* sample, *Onchocerca volvulus* samples and *Loa la* samples. Nonspecific amplification occurred for *Toxoplasma gondii* and *Wuchereria bancrofti*. The observed Tm for these species could not lead to any misidentification with *Plasmodium* spp*.*
